# Robust Distributed High-Dimensional Regression: A Convoluted Rank Approach

**DOI:** 10.3390/e28010119

**Published:** 2026-01-19

**Authors:** Mingcong Wu

**Affiliations:** School of Statistics and Data Science, Southwestern University of Finance and Economics, Chengdu 611130, China; wumingcong@swufe.edu.cn

**Keywords:** robust regression, distributed learning, high dimensions, heavy-tailed errors, non-asymptotic analysis

## Abstract

This paper investigates robust high-dimensional convoluted rank regression in distributed environments. We propose an estimation method suitable for sparse regimes, which remains effective under heavy-tailed errors and outliers, as it does not impose moment assumptions on the noise distribution. To facilitate scalable computation, we develop a local linear approximation algorithm, enabling fast and stable optimization in high-dimensional settings and across distributed systems. Our theoretical results provide non-asymptotic error bounds for both one-round and multi-round communication schemes, explicitly quantifying how estimation accuracy improves with additional communication rounds. Specifically, after a number of communication rounds (logarithmic in the number of machines), the proposed estimator achieves the minimax-optimal convergence rate, up to logarithmic factors. Extensive simulations further demonstrate stable performance across a wide range of error distributions, with accurate coefficient estimation and reliable support recovery.

## 1. Introduction

In the era of big data, the exponential growth in data volume and complexity has made traditional single-machine approaches insufficient for effective statistical modeling. To overcome this challenge, distributed algorithms break down data into manageable chunks and distribute them across multiple computing nodes for parallel processing. This approach greatly improves computational efficiency and scalability, proving essential for handling large-scale data analysis [1,2]. Distributed algorithms are widely used in various statistical learning tasks. For example, Wang et al. [3] studied distributed inference for the linear support vector machine (SVM) in the binary classification task and proposed a multi-round distributed linear-type estimator to achieve computational efficiency in conducting inference for the linear SVM. Chen et al. [4] established a new connection between quantile regression and ordinary linear regression and provided a distributed estimator that is both computationally and communicationally efficient. Chen and Peng [5] explored distributed statistical inference for a general class of statistics, including *U*-statistics and *M*-estimators, in the context of massive data. Additionally, concerns over privacy and regulatory compliance often result in data being stored across multiple institutions, where centralized aggregation is either impractical or prohibited. These challenges have further driven the development of distributed estimation methods that allow for statistical inference without requiring direct access to raw data [6].

A common class of distributed estimation methods follows the one-shot paradigm, where each machine independently computes a local estimator, and the final estimate is obtained through a single round of averaging [1,7,8]. While these methods are attractive due to their simplicity and low communication cost, they often require each machine to hold a sufficiently large number of samples to ensure accurate global estimation. When the local sample size is limited or the number of machines is large, their statistical performance can degrade significantly. To address the limitations of one-shot estimators under limited local sample sizes, Jordan et al. [2] proposed the Communication-Efficient Surrogate Likelihood (CSL) framework. For this approach, each machine computes the gradient of its local loss at a shared parameter value and sends it to a central node, which then constructs a surrogate loss function to approximate the global objective. The estimator is refined iteratively, leading to improved statistical accuracy with logarithmic communication rounds. Inspired by this, several works have extended the CSL method to high-dimensional settings, such as sparse linear models, generalized linear models, quantile regression and modal regression [9,10,11,12,13,14].

In the centralized setting, a substantial body of work has developed robust high-dimensional regression methods with strong theoretical guarantees in the presence of heavy-tailed errors and outliers. Among these methods, Wilcoxon rank regression (also referred to as rank regression) stands out. As introduced by Hettmansperger and McKean [15], the rank regression estimator achieves arbitrarily high relative efficiency under heavy-tailed error distributions while retaining at least 86.4% asymptotic relative efficiency under any symmetric error distribution with finite Fisher information. This combination of robustness and high estimation efficiency makes rank regression a powerful and attractive alternative to standard least squares. Consequently, rank regression has garnered increased attention in recent years. For instance, Wang et al. [16] proposed a tuning-free robust high-dimensional regression procedure based on a simulated tuning parameter, achieving near-oracle performance and strong robustness to heavy-tailed errors. Zhou et al. [17] developed sparse convoluted rank regression by smoothing the rank loss, establishing non-asymptotic properties and presenting an efficient algorithm for high-dimensional settings, in contrast to canonical rank regression. Cai et al. [18] explored statistical inference for high-dimensional convoluted rank regression, deriving estimation error bounds and constructing debiased estimators with valid simultaneous confidence intervals. Building on this line of work, we propose a distributed high-dimensional convoluted rank regression (DCR). Our main contributions are summarized as follows.

- (Methodology). In the CSL framework, we propose a robust, communication-efficient, penalized estimator for high-dimensional linear regression. The proposed procedure does not impose moment assumptions on the error distribution and therefore remains applicable in the presence of heavy-tailed noise and outliers. It is tailored for sparse high-dimensional regimes and delivers accurate coefficient estimation, along with reliable support recovery. Compared with the distributed Huber regression approach proposed by Luo et al. [14], our method does not require the noise to have a finite second moment and avoids the need to tune the two key hyperparameters in the Huber loss. As a result, it is computationally more efficient and easier to implement in practice. Extensive simulation studies in Section 5 demonstrate that the method is stable across a wide range of error distributions and achieves strong empirical performance in both estimation and support recovery.
- (Algorithm). To solve the resulting optimization problem efficiently, we develop a local linear approximation-based algorithm. The algorithm converts the original objective into a sequence of tractable subproblems and can be implemented efficiently in distributed environments, leading to fast and stable computation in high dimensions.
- (Theory). We establish non-asymptotic theoretical guarantees for the proposed estimator under both one-round and multiple-round communication schemes. Specifically, we derive non-asymptotic error bounds characterizing how the accuracy improves as the number of communication rounds increases. After a sufficient number of communication rounds (logarithmic in the number of machines), the distributed estimator attains the minimax convergence rate (up to logarithmic factors).

The rest of the paper is organized as follows. Section 2 introduces the DCR estimator. Section 3 presents an optimization algorithm for the DCR estimator. Section 4 provides a comprehensive theoretical study of the DCR estimator. Numerical results, including simulation studies and real data analysis, are given in Section 5. All technical proofs are provided in Appendix A, Appendix B and Appendix C.

*Notation.* For a positive integer *m*, let [m]={1,…,m}. Denote by $S^{d-1}$, I(·), $e_{d}$ and $I_{d}$ the *d*-dimensional unit sphere, the indicator function, the identity vector and the d×d identity matrix, respectively. For any $m_{1}\times m_{2}$ matrix $H={\left(h_{i,j}\right)}_{m_{1}\times m_{2}}$, let ${∥H∥}_{2}$ denote the spectral norm of matrix H. Write ${∥H∥}_{\infty }=\max_{i\in \left[m_{1}\right],\;j\in \left[m_{2}\right]}\left|h_{i,j}\right|$. Specifically, if $m_{2}=1$, we use ${\left|H\right|}_{1}=\sum_{i=1}^{m_{1}}\left|h_{i,1}\right|$ and ${\left|H\right|}_{2}={\left(\sum_{i=1}^{m_{1}}h_{i,1}^{2}\right)}^{1/2}$ to denote, respectively, the $L_{1}$-norm and $L_{2}$-norm of the $m_{1}$-dimensional vector H. If H is positive semi-definite and $m_{1}=m_{2}$, for any vector $u\in R^{m_{1}}$, let ${\left|u\right|}_{H}={\left|H^{1/2}u\right|}_{2}$. Define the ball $B_{H}\left(r\right)=\{u\in R^{m_{1}}{:|u|}_{H}\leq r\}$ and its translation $B_{H}\left(v,r\right)=v+B_{H}\left(r\right)$ for any r>0 and $v\in R^{m_{1}}$. For a countable set *S*, we use |S| to denote its cardinality. For any two real numbers *a* and *b*, we write a∧b=min(a,b) and a∨b=max(a,b). For two sequences of positive numbers $\left\{a_{n}\right\}$ and $\left\{b_{n}\right\}$, we write $a_{n}≲b_{n}$ or $b_{n}≳a_{n}$ if there exist a positive constant *c* and a large enough integer $n_{0}$ such that $a_{n}/b_{n}\leq c$ for all $n\geq n_{0}$. Write $a_{n}≍b_{n}$ if and only if $a_{n}≲b_{n}$ and $b_{n}≲a_{n}$ hold simultaneously. We use $C,C_{1},\ldots$ to denote some generic positive constants, which may be different in different uses. Let $B\left(r\right)=\{u\in R^{k}:|u{|}_{2}\leq r\}$ be the Euclidean ball with radius *r*. Denote the zero-mean operator (1−E)(ζ)=ζ−E(ζ) for any random matrix ζ.

## 2. Distributed High-Dimensional Convoluted Rank Regression

Suppose that the sample of i.i.d. observations ${\left\{\left(Y_{i},X_{i}\right)\right\}}_{i=1}^{N}$ are generated from the following linear regression model,

$$Y_{i}=X_{i}^{⊤}\beta^{*}+\varepsilon_{i}\;,$$

where ${\left\{Y_{i}\right\}}_{i=1}^{N}$ are one-dimensional responses, ${\left\{\varepsilon_{i}\right\}}_{i=1}^{N}$ are random errors, ${\left\{X_{i}\right\}}_{i=1}^{N}$ are *p*-dimensional random vectors with mean zero and covariance matrix Σ and $\beta^{*}={\left(\beta_{1}^{*},\ldots ,\beta_{p}^{*}\right)}^{⊤}\in R^{p}$ is the unknown parameter of interest.

We begin by introducing the global convoluted rank regression, which forms the basis for our distributed extension. Given a sample of observations ${\left\{\left(Y_{i},X_{i}\right)\right\}}_{i=1}^{N}$, the objective is to estimate the regression coefficient vector $\beta^{*}\in R^{p}$ by minimizing a smoothed version of the canonical rank loss. Following the formulation in Zhou et al. [17], we define the convolution-based rank loss as

$${\hat{L}}_{h}\left(\beta \right)=\frac{1}{N(N-1)}\sum_{i=1}^{N}\sum_{j\neq i}L_{h}\left(\left(Y_{i}-X_{i}^{⊤}\beta \right)-\left(Y_{j}-X_{j}^{⊤}\beta \right)\right),$$

where $L_{h}\left(\cdot \right)$ is a kernel-smoothed convex loss function defined as

$$L_{h}\left(u\right)=\int_{-\infty }^{\infty }\left|u-v\right|K_{h}\left(v\right)\;dv\;,$$

where $K_{h}\left(\cdot \right)=h^{-1}K\left(\cdot /h\right)$ with K(·) being the kernel function and h>0 being the bandwidth. We define the population parameter as

$$\beta_{h}^{*}\in \underset{\beta \in R^{p}}{\mathrm{argmin}}E\left\{{\hat{L}}_{h}\left(\beta \right)\right\}\;.$$

By Theorem 1 of Zhou et al. [17], we have $\beta_{h}^{*}=\beta^{*}$ for any h>0. Thus, in high-dimensional settings (p≫n), we introduce the global penalized convoluted rank regression estimator for $\beta^{*}$ with entrywise regularizations as follows:

$$\hat{\beta }\in \underset{\beta \in R^{p}}{\mathrm{argmin}}\left\{{\hat{L}}_{h}\left(\beta \right)+\sum_{j=1}^{p}p_{\lambda }\left(\right|\beta_{j}\left|\right)\right\}\;,$$

where $\beta_{j}$ is the *j*-th element of $\beta \in R^{p}$ and $p_{\lambda }\left(\cdot \right)$ is a penalty function with λ>0 being a tuning parameter.

In the distributed setting, assume that the entire dataset ${\left\{\left(Y_{i},X_{i}\right)\right\}}_{i=1}^{N}$ is stored across *m* node machines: one central machine and m−1 local machines connected to the central machine. For j∈[m], the *j*-th machine stores a subsample of $n_{j}$ observations, denoted by ${\left\{\left(Y_{i},X_{i}\right)\right\}}_{i\in I_{j}}$, where $I_{j}$ are disjoint index sets such that $\cup_{j=1}^{m}I_{j}=\left[N\right]$ and $N=\sum_{j=1}^{m}\left|I_{j}\right|=\sum_{j=1}^{m}n_{j}$. Without loss of generality, we assume that $n_{1}=\cdots =n_{m}=n$, so that N=nm. We refer to *n* as the local sample size.

Given bandwidth h>0, we define the local convoluted rank loss as

(1) $$\begin{array}{c} {\hat{L}}_{j,h}\left(\beta \right)=\frac{1}{n(n-1)}\sum_{i\in I_{j}}\sum_{k\in I_{j}:\;k\neq i}L_{h}\left(\left(Y_{i}-X_{i}^{⊤}\beta \right)-\left(Y_{k}-X_{k}^{⊤}\beta \right)\right)\;. \end{array}$$

Starting with an initial estimator ${\hat{\beta }}^{\left(0\right)}$ for $\beta^{*}$, we define the surrogate loss as

(2) $${\hat{L}}_{h}^{\left(t\right)}\left(\beta \right)={\hat{L}}_{1,h}\left(\beta \right)-\langle \nabla {\hat{L}}_{1,h}\left({\hat{\beta }}^{\left(t-1\right)}\right)-\nabla {\hat{L}}_{h}\left({\hat{\beta }}^{\left(t-1\right)}\right),\beta \rangle \;.$$

We allow the parametric dimension *p* to be much larger than the local sample size *n*. Denote by $S=\mathrm{supp}\left(\beta^{*}\right)=\left\{j\in \left[p\right]:\beta_{j}^{*}\neq 0\right\}$ the true active set. Hence, $\beta^{*}$ is *s*-sparse with s=|S|. At iteration t∈[T] for integer T>0, the first machine solves the following minimization problem:

(3) $$\begin{array}{c} {\hat{\beta }}^{\left(t\right)}\in \underset{\beta \in R^{p}}{\mathrm{argmin}}\left\{{\hat{L}}_{h}^{\left(t\right)}\left(\beta \right)+\sum_{j=1}^{p}p_{\lambda_{t}}\left(\right|\beta_{j}\left|\right)\right\}\;, \end{array}$$

where $\lambda_{t}>0$ is a tuning parameter. We choose ${\hat{\beta }}^{\left(0\right)}$ to be the local penalized convoluted rank regression estimator such that

$$\begin{array}{c} {\hat{\beta }}^{\left(0\right)}\in \underset{\beta \in R^{p}}{\mathrm{argmin}}\left\{{\hat{L}}_{1,h}\left(\beta \right)+\sum_{j=1}^{p}p_{\lambda_{0}}\left(\right|\beta_{j}\left|\right)\right\}\;. \end{array}$$

In the next section, we will propose an optimization algorithm for solving (3).

## 3. Optimization Algorithm

In this section, we focus on the iterations after the (t−1)-th communication round. At the (k+1)-th iteration, we use the local linear approximation (LLA) algorithm to update the estimate by solving (3) as follows:

$$\begin{array}{c} {\hat{\beta }}_{k+1}^{\left(t\right)}\in \underset{\beta \in R^{p}}{\mathrm{argmin}}\left\{{\hat{L}}_{h}^{\left(t\right)}\left(\beta \right)+\sum_{j=1}^{p}p_{\lambda_{t}}^{\prime }\left(\right|\beta_{k,j}^{\left(t\right)}\left|)\right|\beta_{j}|\right\}\;, \end{array}$$

where $\beta_{k}^{\left(t\right)}={\left(\beta_{k,1}^{\left(t\right)},\ldots ,\beta_{k,p}^{\left(t\right)}\right)}^{⊤}$ is the previous estimate in the *k*-th iteration. Specifically, in a coordinate-wise manner, we update the *l*-th coordinate ${\hat{\beta }}_{l,k+1}^{\left(t\right)}$ by minimizing

$$\begin{array}{cc} F({\hat{\beta }}_{l,k+1}^{\left(t\right)}\;|\;\beta_{k}^{\left(t\right)})= & \;\frac{1}{n(n-1)}\sum_{i\in I_{1}}\sum_{j\in I_{1}:j\neq i}L_{h}\left(\omega_{i,j}-\left(X_{i,l}-X_{j,l}\right)\left({\hat{\beta }}_{l,k+1}^{\left(t\right)}-\beta_{l,k}^{\left(t\right)}\right)\right)\\ \; & \;\;\;-\left\{\nabla_{l}{\hat{L}}_{1,h}\left({\hat{\beta }}^{\left(t-1\right)}\right)-\nabla_{l}{\hat{L}}_{h}\left({\hat{\beta }}^{\left(t-1\right)}\right)\right\}{\hat{\beta }}_{l,k+1}^{\left(t\right)}+p_{\lambda_{t}}^{\prime }\left(\right|\beta_{l,k}^{\left(t\right)}\left|)\right|{\hat{\beta }}_{l,k+1}^{\left(t\right)}|\;, \end{array}$$

where ${\hat{\beta }}^{\left(t-1\right)}$ is defined in (3) and $\omega_{i,j}=Y_{i}-Y_{j}-{\left(X_{i}-X_{j}\right)}^{⊤}{\hat{\beta }}_{k}^{\left(t\right)}$. We let

(4) $$\begin{array}{c} \upsilon_{l,1}=-\frac{1}{n(n-1)}\sum_{i\in I_{1}}\sum_{j\in I_{1}:\;i\neq j}L_{h}^{\prime }\left(\omega_{i,j}\right)\left(X_{i,l}-X_{j,l}\right)\;, \end{array}$$

(5) $$\begin{array}{c} \upsilon_{l,2}=\frac{1}{n(n-1)}\sum_{i\in I_{1}}\sum_{j\in I_{1}:\;i\neq j}{\left(X_{i,l}-X_{j,l}\right)}^{2}\;, \end{array}$$

(6) $$\begin{array}{c} \upsilon_{l,3}=\nabla_{l}{\hat{L}}_{1,h}\left({\hat{\beta }}^{\left(t-1\right)}\right)-\nabla_{l}{\hat{L}}_{h}\left({\hat{\beta }}^{\left(t-1\right)}\right)\;. \end{array}$$

By Lemma 1 of Zhou et al. [17], we can construct a quadratic majorization function for $F({\hat{\beta }}_{l,k+1}^{\left(t\right)}\;|\;\beta_{k}^{\left(t\right)})$:

$$\begin{array}{cc} G({\hat{\beta }}_{l,k+1}^{\left(t\right)}\;|\;\beta_{k}^{\left(t\right)})= & \;\frac{1}{n(n-1)}\sum_{i\in I_{1}}\sum_{j\neq i\in I_{1}}L_{h}\left(\omega_{i,j}\right)+\upsilon_{l,1}\left({\hat{\beta }}_{l,k+1}^{\left(t\right)}-\beta_{l,k}^{\left(t\right)}\right)\\ \; & \;+\frac{k_{6}\upsilon_{l,2}}{h}{\left({\hat{\beta }}_{l,k+1}^{\left(t\right)}-\beta_{l,k}^{\left(t\right)}\right)}^{2}-\upsilon_{l,3}{\hat{\beta }}_{l,k+1}^{\left(t\right)}+p_{\lambda_{t}}^{\prime }\left(\right|\beta_{l,k}^{\left(t\right)}\left|)\right|{\hat{\beta }}_{l,k+1}^{\left(t\right)}|\;. \end{array}$$

Hence, we can update ${\hat{\beta }}_{l,k+1}^{\left(t\right)}$ using the minimizer of $G({\hat{\beta }}_{l,k+1}^{\left(t\right)}\;|\;\beta_{k}^{\left(t\right)})$:

$$\begin{array}{c} {\hat{\beta }}_{l,k+1}^{\left(t\right)}=\mathrm{sgn}\left(\beta_{l,k}^{\left(t\right)}-\frac{h(\upsilon_{l,1}-\upsilon_{l,3})}{2\upsilon_{l,2}k_{6}}\right)\max\left\{|\beta_{l,k}^{\left(t\right)}-\frac{h(\upsilon_{l,1}-\upsilon_{l,3})}{2\upsilon_{l,2}k_{6}}|-\frac{hp_{\lambda_{t}}^{\prime }\left(\right|\beta_{l,k}^{\left(t\right)}\left|\right)}{2\upsilon_{l,2}k_{6}},0\right\}\;. \end{array}$$

For tuning parameter $\lambda_{t}$, we set $\lambda_{t}=\lambda_{1}$ for all t∈[T]. To select an appropriate value of $\lambda_{1}$, we propose a high-dimensional Bayesian information criterion (HBIC) in the distributed setting, which is defined as

$$\begin{array}{c} \mathrm{HBIC}\left(\lambda \right)=\;\log\;{\hat{L}}_{h}^{\left(1\right)}\left({\hat{\beta }}^{\left(1\right)}\right)+\frac{|M_{\lambda }|\left(\;\log\;\;\log\;n\right)\;\log\;p}{n}\;, \end{array}$$

where $M_{\lambda }=\left\{j\in \left[p\right]:{\hat{\beta }}_{j}^{\left(1\right)}\neq 0\right\}$ with ${\hat{\beta }}_{j}^{\left(1\right)}$ being the *j*-th element of ${\hat{\beta }}^{\left(1\right)}$. Compared to cross-validation, selecting $\lambda_{1}$ by minimizing the HBIC eliminates the need to split the data or repeatedly evaluate test error across multiple sub-datasets, thereby reducing computational cost while maintaining theoretical interpretability. Specifically, we compute the solution path over a decreasing grid ${\left\{\lambda_{1,\ell }\right\}}_{\ell =1}^{L}$, evaluating the HBIC for each $\lambda_{1,\ell }$ and choosing the minimizer. In implementation, we first compute an initial penalty level $\lambda_{1,1}$ similarly to Zhou et al. [17], using only the local data on the first machine to avoid extra communication, which is represented as follows:

$$\begin{array}{c} \lambda_{1,1}=|\frac{1}{n(n-1)}\sum_{i\in I_{1}}\sum_{j\in I_{1},j\neq i}L_{h}^{\prime }\left(Y_{i}-Y_{j}\right)\left(X_{i}-X_{j}\right){|}_{\infty }\;. \end{array}$$

We then form a decreasing geometric grid $\lambda_{\ell }=0.1^{1/(L-1)}\lambda_{\ell -1}$ for ℓ>1. In our experiments, we set L=50. We summarize this procedure in Algorithm 1, which is based on Algorithm 2.
**Algorithm 1** Estimation procedure for DCR**Require:** (i) data batches ${\left\{\left(Y_{i},X_{i}\right)\right\}}_{i\in I_{j}}$, j∈[m], stored on *m* local machines; (ii) initialization ${\hat{\beta }}^{\left(0\right)}$; (iii) bandwidth *h*, regularization parameter λ>0, and communication times *T*. 1:**for** t∈[T] **do** 2:  Compute local gradient $\nabla {\hat{L}}_{1,h}\left({\hat{\beta }}^{\left(t-1\right)}\right)$ and global gradient $\nabla {\hat{L}}_{h}\left({\hat{\beta }}^{\left(t-1\right)}\right)$; 3:  Use Algorithm 2 to compute ${\hat{\beta }}^{\left(t\right)}$. 4:**end for****Ensure:** ${\hat{\beta }}^{\left(T\right)}$.

**Algorithm 2** Local linear approximation (LLA) algorithm for solving (3)
**Require:** (i) local data ${\left\{\left(Y_{i},X_{i}\right)\right\}}_{i\in I_{1}}$; (ii) initialization ${\hat{\beta }}^{\left(t-1\right)}$; (iii) local gradient $\nabla {\hat{L}}_{1,h}\left({\hat{\beta }}^{\left(t-1\right)}\right)$; (iv) global gradient $\nabla {\hat{L}}_{h}\left({\hat{\beta }}^{\left(t-1\right)}\right)$; (v) bandwidth *h* and (vi) regularization parameter λ>0.
 1:Initial $\bar{\beta }\leftarrow {\hat{\beta }}^{\left(t-1\right)}$. 2:
**repeat**
 3:  **for** l∈[p] **do** 4:    Compute $\upsilon_{l,1}$, $\upsilon_{l,2}$ and $\upsilon_{l,3}$ for l∈[p] by (4), (5) and (6), respectively. 5:    Compute$$\begin{array}{c} {\tilde{\beta }}_{l}=\mathrm{sign}\left({\bar{\beta }}_{l}-\frac{h(\upsilon_{l,1}-\upsilon_{l,3})}{2\upsilon_{l,2}k_{6}}\right)\max\left\{|{\bar{\beta }}_{l}-\frac{h(\upsilon_{l,1}-\upsilon_{l,3})}{2\upsilon_{l,2}k_{6}}|-\frac{hp^{\prime }\left(\right|{\bar{\beta }}_{l}\left|\right)}{2\upsilon_{l,2}k_{6}},0\right\}\;. \end{array}$$ 6:    Let $d_{l}={\tilde{\beta }}_{l}-{\bar{\beta }}_{l}$ and set ${\bar{\beta }}_{l}\leftarrow {\tilde{\beta }}_{l}$. 7:  **end for** 8:**until** $\max_{l\in [p]}d_{l}^{2}<10^{-7}$.
**Ensure:**  ${\hat{\beta }}^{\left(t\right)}={\left({\tilde{\beta }}_{1},\ldots ,{\tilde{\beta }}_{p}\right)}^{⊤}$.


## 4. Theoretical Results

In this section, we investigate the theoretical properties for the DCR estimator proposed in Section 2. Let $\Sigma =E(XX^{⊤})$ and $Z=\Sigma^{-1/2}X$. Denote by g(·) the probability density function of $\varepsilon_{i}-\varepsilon_{j}$ conditional on $X_{i}$ and $X_{j}$. To establish the theoretical guarantees, we need the following regularity assumptions.

**Assumption** **1.** *There exist universal constants $k_{1},k_{2},k_{3}>0$ such that* (i) *$\max_{i\in [N],j\in [p]}\left|X_{i,j}\right|\leq k_{1}$*; (ii) *$\sup_{u\in S^{p-1}}E\left\{{\left(u^{⊤}Z\right)}^{4}\right\}\leq k_{2}$*; *and* (iii) *the eigenvalues of matrix* Σ *are bounded above by $k_{3}$ and below by $k_{3}^{-1}$.*

**Assumption** **2.** *There exist universal constants $k_{4},k_{5}>0$ such that* (i) *$\left|g\left(x\right)-g\left(y\right)\right|\leq k_{4}\left|x-y\right|$ for all x,y∈R and* (ii) *$k_{5}^{-1}\leq g\left(0\right)\leq k_{5}$.*

**Assumption** **3.** *The kernel function K(·) satisfies that K(v)≥0 and K(−v)=K(v) for any v∈R, $\sup_{v\in R}K^{\prime }\left(v\right)<\infty$, and $\int_{R}K\left(v\right)\;dv=1$. There exist universal constants $k_{6},k_{7}>0$ such that $\sup_{v\in R}K\left(v\right)<k_{6}$ and $\inf_{v\in [-1,1]}K\left(v\right)=k_{7}$.*

**Assumption** **4.** *Suppose that* (i) *the penalty function $p_{\lambda }\left(\cdot \right)$ satisfies $p_{\lambda }\left(0\right)=0$ and $p_{\lambda }\left(t\right)=p_{\lambda }\left(-t\right)$ for all t∈R*; (ii) *$p_{\lambda }\left(\cdot \right)$ is nondecreasing on the nonnegative real line*; (iii) *$p_{\lambda }\left(t\right)/t$ is nonincreasing for t>0*; (iv) *$p_{\lambda }\left(\cdot \right)$ is differentiable for all t≠0 and has a right derivative at t=0 with $\lim_{t\to 0^{+}}p_{\lambda }^{\prime }\left(t\right)=k_{8}\lambda$ for universal constant $k_{8}>0$*; *and* (v) *there exists $0<\mu <7/(5k_{3})$ such that $p_{\lambda ,\mu }\left(t\right)=p_{\lambda }\left(t\right)+\mu t^{2}/2$ is convex.*

Assumptions 1–3 are standard in high-dimensional convoluted rank regression, as discussed in, e.g., Zhou et al. [17]. Assumption 4 is standard in the literature on nonconvex penalized *M*-estimation and has been used in prior works such as Loh and Wainwright [19], Loh [20], Loh and Wainwright [21]. These assumptions ensure basic regularity properties (e.g., symmetry, monotonicity, local differentiability and a localized convexification) and are satisfied by a broad class of nonconvex penalties, including SCAD [22] and MCP [23].

Define the event $E_{0}\left(r_{0}\right)=\left\{{\hat{\beta }}^{\left(0\right)}\in B_{\Sigma }\left(\beta^{*},r_{0}\right)\cap \Lambda \right\}$ for some $r_{0}\geq 0$, where $\Lambda =\{\beta \in R^{p}:|\beta -\beta^{*}{|}_{1}\leq 4s^{1/2}|\beta -\beta^{*}{|}_{\Sigma }\}$ is an $\ell_{1}$-cone. Since we are interested in cases where the penalty may be nonconvex, we include the side condition ${\left|\beta \right|}_{1}\leq R$ for R>0 in (3) in order to guarantee the existence of local/global optima. Theorem 1 gives the convergence rate of the estimator after the first iteration under the norm ${\left|\cdot \right|}_{\Sigma }$, whose proof is given in Appendix A.

**Theorem** **1.** *Let Assumptions* 1–4 *hold. Select $\lambda_{1}≍r_{0}{\left(s\;\log\;p/n\right)}^{1/2}+{\left(\;\log\;p/N\right)}^{1/2}$, h=O(1) and $R≍{\left(n/\;\log\;p\right)}^{1/2}$. Restricted on the event $E_{0}\left(r_{0}\right)$, it holds with probability at least $1-3p^{-1}$ that $\left\{{\hat{\beta }}^{\left(1\right)}\in \Lambda \right\}$ and*

(7) $$\begin{array}{c} |{\hat{\beta }}^{\left(1\right)}-\beta^{*}{|}_{\Sigma }≲\frac{r_{0}s{\left(\;\log\;p\right)}^{1/2}}{n^{1/2}}+\frac{{\left(s\;\log\;p\right)}^{1/2}}{N^{1/2}} \end{array}$$

*provided that n≫logp.*

**Remark** **1.** *Theorem 1 illustrates that a single round of communication among machines can substantially improve the accuracy of the initial estimator ${\hat{\beta }}^{\left(0\right)}$, which is obtained from local computation based on only n samples. Specifically, ${\hat{\beta }}^{\left(1\right)}$ is computed by solving a regularized surrogate problem (3) with t=1, which incorporates both the local loss function ${\hat{L}}_{1,h}\left(\beta \right)$ and the global gradient $\nabla {\hat{L}}_{h}\left({\hat{\beta }}^{\left(0\right)}\right)$, effectively integrating information from the entire dataset of size N=mn. The error bound on the right hand side of (7) demonstrates the gain from global information sharing: the first term reduces the estimation error related to limited local sample size n, while the second term decays with the total sample size N, enabling ${\hat{\beta }}^{\left(1\right)}$ to reach the efficiency of a global estimator.*

Theorem 2 gives the convergence rate of the estimator after the *t*-th iteration under the norm ${\left|\cdot \right|}_{\Sigma }$, whose proof is given in Appendix B.

**Theorem** **2.** *Let Assumptions 1–4 hold. Select h=O(1), $R≍{\left(n/\;\log\;p\right)}^{1/2}$ and $\lambda_{t}≍{\left(\;\log\;p/N\right)}^{1/2}+s^{(2t+1)/2}{\left(\;\log\;p/n\right)}^{(t+1)/2}$. Then, it holds with probability at least $1-3\left(t+1\right)p^{-1}$ that*

(8) $$\begin{array}{c} |{\hat{\beta }}^{\left(t\right)}-\beta^{*}{|}_{\Sigma }≲\frac{s^{(2t+1)/2}{\left(\;\log\;p\right)}^{(t+1)/2}}{n^{(t+1)/2}}+\frac{{\left(s\;\log\;p\right)}^{1/2}}{N^{1/2}} \end{array}$$

*provided that $n\gg s^{2}\;\log\;p$.*

**Remark** **2.** *Theorem 2 highlights the effectiveness of multiple rounds of communication in distributed learning with penalized convoluted rank loss. On the right hand side of (8), the first term decays exponentially with the number of communication rounds t, while the second term matches the optimal rate of global convoluted rank regression with access to the full dataset of size N. Notably, when t≍logm, the influence of the initial estimator ${\hat{\beta }}^{\left(0\right)}$, which only uses local data, is effectively eliminated. As a result, the final estimator ${\hat{\beta }}^{\left(t\right)}$ achieves the same efficiency as one that directly solves the full-data problem, but without the need to collect all N samples on a single machine. This demonstrates that the proposed multi-round distributed procedure enables communication-efficient optimality, and only a logarithmic number of machines are needed to match the convergence rate of global learning.*

## 5. Numerical Studies

### 5.1. Monte Carlo Examples

We consider three methods for comparison with our proposed method: (i) the global convoluted rank regression (Global CR) estimator [17] that uses all the available N=mn observations; (ii) the divide-and-conquer CR (DC-CR) estimator, which is based on averaging *m* local CR estimators; and (iii) the local convoluted rank regression (Local CR). A regularization parameter λ is included in all methods, and its value is selected by minimizing the corresponding HBIC. To measure the sparsity recovery performance, we calculated the F1 score:   

$$F1=\frac{2(\mathrm{TP})}{2(\mathrm{TP})+\mathrm{FP}+\mathrm{FN}}\;,$$

where TP, FP and FN denote the numbers of true positives, false positives and false negatives, respectively. To measure estimation accuracy, we calculate the square root of the mean squared error (RMSE).

We generate data vectors ${\left\{\left(Y_{i},X_{i}\right)\right\}}_{i=1}^{N}$ from a heteroscedastic model $Y=X^{⊤}\beta^{*}+(\sqrt{3}|\beta^{*}{{|}_{2}^{2})}^{-1}{\left(X^{⊤}\beta^{*}\right)}^{2}\varepsilon$, where $c=\sqrt{3}{\left|\beta^{*}\right|}_{2}^{2}$, $\beta^{*}={\left(\sqrt{3},\sqrt{3},\sqrt{3},\sqrt{3},\sqrt{3},0,0,\ldots ,0\right)}^{⊤}\in R^{p}$, and X is independently generated from N(0,Σ) with $\Sigma_{i,j}={\left(0.5^{\left|i-j\right|}\right)}_{p\times p}$. We consider that ε is independently generated from (1) standard normal distribution ε∼N(0,1); (2) *t* distribution ε∼t(2); (3) standard Cauchy distribution ε∼Cauchy(0,1); and (4) mixture normal distribution ε∼0.75N(0,1)+0.25N(0,100). We set p=400, n=200, m∈{10,20,30,40,50} and the number of communication rounds T=⌈logm⌉. In practice, we recommend an early-stopping strategy for *T* similar to that proposed by Zhang et al. [1]: communication iterations can be terminated when the gradient norm of the loss Function (2) drops below a predefined threshold.

We adopt the Epanechnikov kernel $K\left(u\right)=3/4\cdot \left(1-u^{2}\right)I\left(-1\leq u\leq 1\right)$ and construct the loss function as

$$\begin{array}{c} L_{h}\left(u\right)=\left\{\begin{array}{cc} u\;, & u\geq h\;,\\ \frac{3u^{2}}{4h}-\frac{u^{4}}{8h^{3}}+\frac{3h}{8}\;, & -h<u<h\;,\\ u\;, & u\leq -h\;. \end{array}\right. \end{array}$$

In Algorithm 1, the bandwidth *h* is used only to smooth the rank loss. Importantly, this smoothing does not change the target parameter: by Theorem 1 of Zhou et al. [17], the population minimizer satisfies $\beta_{h}^{*}=\beta^{*}$ for any h>0. Therefore, *h* mainly affects numerical smoothness and finite-sample constants rather than the estimator. Our theory only requires *h* to be of constant order, i.e., h=O(1). In practice, we recommend a simple default choice of h=1. For regularization, we employ the SCAD penalty, which is given by

$$\begin{array}{c} p_{\lambda }\left(|t|\right)=\left\{\begin{array}{cc} \lambda |t|\;, & 0\leq |t|<\lambda \;,\\ \frac{a\lambda |t|-(t^{2}+\lambda^{2})/2}{a-1}\;, & \lambda \leq |t|\leq a\lambda \;,\\ \frac{\left(a+1\right)\lambda^{2}}{2}\;, & |t|>a\lambda \;, \end{array}\right. \end{array}$$

for some a>2. The default choice of parameter *a* is 3.7.

The simulation results, summarized in Table 1, show that the proposed DCR method achieves the best overall performance across all settings. It consistently yields the lowest RMSE and the highest F1 scores, indicating both accurate estimation and reliable support recovery. The DC-CR estimator, while comparable to DCR in terms of RMSE, suffers from significantly lower F1 scores, particularly under Cauchy and contaminated distributions, due to unstable variable selection. These results highlight the advantage of DCR in combining robustness, sparsity and distributed efficiency.

### 5.2. Real Data Example: LGBT Tweets

We illustrate our proposed method on a real-world dataset of tweets related to LGBTQ topics, collected using the keyword “LGBT” over the period from 21 August to 26 August 2022 (the dataset is publicly available at https://www.kaggle.com/datasets/vencerlanz09/lgbt-tweets (accessed on 20 April 2025)). The goal of this analysis is to identify linguistically salient words that have a significant impact on the like count of each tweet.

**Figure 1:**
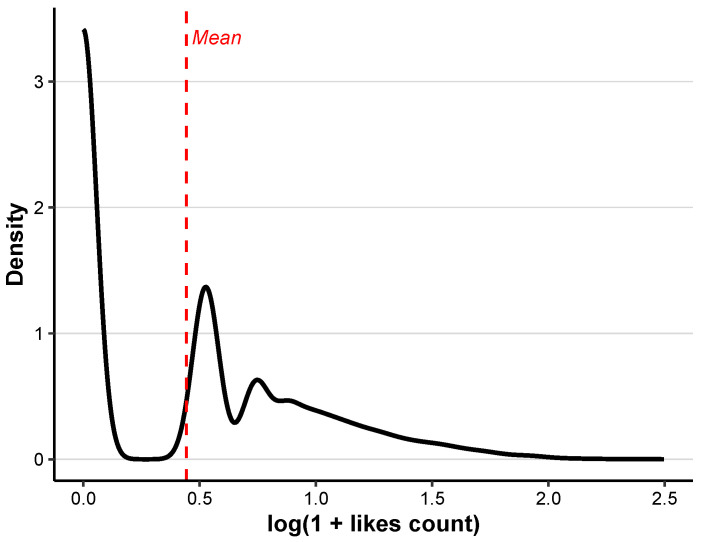
Probability density of log(1+likescount).

Each observation in our study represents a single post. Our initial sample consists of a total of N=32,456 tweets, each containing the keyword “LGBT”. For each post, we record the number of likes it received, denoted by $Y_{i}$, which serves as the outcome variable. The original like counts $Y_{i}$ exhibit substantial variation, with most tweets receiving only a few likes and a small number garnering extremely high engagement. To address this scale disparity and stabilize the variance, we apply the transformation $\;\log\;(1+Y_{i})$. As shown in Figure 1, the transformed variable retains a heavy-tailed distribution, indicating that the imbalance in user attention persists even after logarithmic scaling.

To construct the covariates, we preprocess the tweet text using Python 3.11.10 libraries, including spaCy for tokenization, lemmatization and part-of-speech filtering, and CountVectorizer for feature extraction. During preprocessing, we remove punctuation, stop words, auxiliary verbs, adverbs and informal abbreviations. After cleaning, we apply a binary bag-of-words encoding based on the 500 most frequently used informative words in the corpus. Specifically, the *j*-th coordinate $X_{ij}$ of the covariate vector $X_{i}$ equals 1 if the *j*-th word in the vocabulary appears in the *i*-th tweet, and 0 otherwise. Posts that do not contain any of these 500 words are excluded from the analysis. After filtering, we retain a final sample of *N* = 31,644 observations. The data is randomly distributed across 100 machines, with an average of 316 data points per machine. The number of iterations is set to T=5.

From Table 2, we observe that several keywords show strong positive associations with the number of likes, such as “character”, “queer” and “let”. These terms frequently appear in contexts emphasizing representation, identity affirmation or emotional solidarity (e.g., “queer character in media” and “let people love who they love”). Such expressions typically convey inclusive and positive sentiments, which tend to resonate with audiences and encourage affirmative responses in the form of likes.

In contrast, negatively weighted terms such as “groom”, “ban” and “loom” are often embedded in narratives involving controversy, fear or stigmatization. For instance, tweets referencing “ban LGBT education” or “danger looms” may attract attention but are less likely to elicit endorsement via likes, as users may refrain from visibly supporting content that is perceived as divisive or politically sensitive. Additionally, high-frequency yet semantically broad words such as “right” and “school” demonstrate weak or even negative associations with like counts. In particular, “school” frequently appears in tweets related to educational debates (e.g., “LGBT in schools” and “teaching gender identity”), which are often contentious. The polarizing nature of such discussions may lead users to engage cautiously or avoid expressing approval through likes.

Overall, the keywords selected by our proposed method highlight the crucial role of emotional valence and semantic framing in shaping engagement behavior. While positively framed language that conveys support, identity and collective belonging tends to promote user interaction, negatively charged or controversial expressions are less likely to generate positive engagement, despite their potential visibility.

## 6. Discussion

In this paper, we study high-dimensional linear regression in distributed settings and develop a DCR framework to mitigate the severe performance degradation of least-squares-based distributed procedures under heavy-tailed errors and outliers. Methodologically, the proposed approach integrates a convolution-smoothed rank loss into a communication-efficient surrogate-likelihood paradigm: at each communication round, a tractable surrogate objective is constructed by combining local empirical losses with a global gradient correction, and the central node solves a sparsity-regularized problem with nonconvex penalties (e.g., SCAD/MCP) to aggregate information without transferring raw data. To enable scalable computation, we further devise an optimization strategy based on the local linear approximation (LLA) method, reducing the nonconvex regularized estimation to a sequence of efficiently solvable subproblems. Additionally, an HBIC-based tuning procedure is provided to alleviate the computational burden of cross-validation in large-scale distributed regimes.

On the theoretical side, we establish non-asymptotic error bounds for both one-shot and multi-round communication schemes, explicitly characterizing how additional communication improves statistical accuracy. In particular, a single communication round already yields a substantial correction over purely local estimators, while the multi-round procedure exhibits a rapid contraction of the “local-sample-limitation” term in the error bound. When the number of communication rounds reaches a logarithmic order in the number of machines, the resulting estimator attains (up to logarithmic factors) the optimal convergence rate comparable to its centralized counterpart, thereby achieving communication-efficient statistical optimality.

Extensive experiments corroborate the robustness and effectiveness of the proposed method. Across a broad collection of noise distributions, DCR delivers stable performance in terms of estimation accuracy and support recovery. In a real-data application, DCR identifies interpretable keywords and yields effect estimates with coherent directions, illustrating its practical utility for large-scale high-dimensional problems with non-Gaussian noise.

Several directions merit further investigation. First, it would be valuable to extend the proposed framework to broader model classes (e.g., generalized linear or semiparametric models) and to formally analyze robustness and computational behavior under more complex data structures such as dependence, nonstationarity or distributional drift. Second, building on the current estimation theory, developing feasible distributed inference procedures along with a principled characterization of the trade-offs among inferential accuracy, communication cost and computation would substantially broaden the applicability of method. Third, for partially trusted distributed environments, integrating DCR with Byzantine-resilient aggregation or fault-tolerant mechanisms and establishing statistical consistency and worst-case error guarantees in the presence of adversarially corrupted worker messages would enhance both the security and reliability of the method in real-world deployments. We leave these considerations for future research.

## Figures and Tables

**Table 1:** Comparison of Local CR, DC-CR, DCR and Global CR.

	N(0,1)									
	m=10		m=20		m=30		m=40		m=50	
Model	RMSE	F1	RMSE	F1	RMSE	F1	RMSE	F1	RMSE	F1
Local CR	0.010	1.000	0.010	1.000	0.010	1.000	0.010	1.000	0.010	1.000
DC-CR	0.003	1.000	0.002	0.997	0.002	0.997	0.001	0.995	0.001	0.995
DCR	0.003	1.000	0.002	1.000	0.002	1.000	0.002	1.000	0.001	1.000
Global CR	0.004	1.000	0.003	1.000	0.002	1.000	0.002	1.000	0.002	1.000
	t(2)									
	m=10		m=20		m=30		m=40		m=50	
Model	RMSE	F1	RMSE	F1	RMSE	F1	RMSE	F1	RMSE	F1
Local CR	0.012	0.999	0.012	0.999	0.012	0.999	0.012	0.999	0.012	0.999
DC-CR	0.004	0.977	0.003	0.967	0.003	0.953	0.002	0.940	0.002	0.927
DCR	0.004	0.999	0.003	0.999	0.002	0.999	0.002	0.999	0.002	0.999
Global CR	0.005	1.000	0.004	1.000	0.003	1.000	0.002	1.000	0.002	1.000
	Cauchy(0,1)									
	m=10		m=20		m=30		m=40		m=50	
Model	RMSE	F1	RMSE	F1	RMSE	F1	RMSE	F1	RMSE	F1
Local DCR	0.016	0.983	0.016	0.983	0.016	0.983	0.016	0.983	0.016	0.983
DC-CR	0.009	0.872	0.007	0.760	0.006	0.683	0.005	0.610	0.005	0.556
DCR	0.005	0.985	0.004	0.985	0.004	0.985	0.003	0.985	0.003	0.985
Global CR	0.007	1.000	0.004	1.000	0.004	1.000	0.003	1.000	0.003	1.000
	0.75N(0,1)+0.25N(0,100)									
	m=10		m=20		m=30		m=40		m=50	
Model	RMSE	F1	RMSE	F1	RMSE	F1	RMSE	F1	RMSE	F1
Local CR	0.018	0.967	0.018	0.967	0.018	0.967	0.018	0.967	0.018	0.967
DC-CR	0.009	0.799	0.006	0.690	0.005	0.591	0.004	0.521	0.004	0.460
DCR	0.006	0.977	0.004	0.977	0.004	0.977	0.004	0.977	0.004	0.977
Global CR	0.007	1.000	0.004	1.000	0.003	1.000	0.003	1.000	0.003	1.000

**Table 2:** Estimation result for LGBT tweets data.

Word	Count	${\hat{\beta }}_{j}$
loom	139	−0.443
character	735	0.335
queer	680	0.214
groom	130	−0.117
ban	689	−0.109
let	400	0.088
write	200	0.070
school	806	−0.051
right	2323	−0.027
force	460	−0.027
future	117	−0.022

## Data Availability

The data presented in this study are openly available in LGBT Tweets at https://www.kaggle.com/datasets/vencerlanz09/lgbt-tweets (accessed on 20 April 2025).

## Appendix A. Proof of Theorem 1

**Proof of** **Theorem 1.** We let

$$\begin{array}{c} A\left(r,l,\kappa \right)=\left\{\frac{\langle \nabla {\hat{L}}_{h}\left(\beta \right)-\nabla {\hat{L}}_{h}\left(\beta^{*}\right),\beta -\beta^{*}\rangle }{|\beta -\beta^{*}{|}_{\Sigma }^{2}}\geq \kappa \;,\mathrm{for}\;\mathrm{all}\;\beta -\beta^{*}\in B_{\Sigma }\left(r\right)\cap C_{\Sigma }\left(l\right)\right\} \end{array}$$

with $C_{\Sigma }\left(l\right)=\{\delta \in R^{p}{:|\delta |}_{1}\leq {l|\delta |}_{\Sigma }\}$. In order to proof Theorem 1, we need the following lemmas, whose proof are given in Appendix C.1, Appendix C.2 and Appendix C.3, respectively. **Lemma** **A1.** *Let Assumptions* 1 *and* 3 *hold. Then, it holds that*

$$\begin{array}{c} \underset{\beta -\beta^{*}\in B_{\Sigma }\left(r\right)\cap \Lambda }{\sup}{\left|\left(1-E\right)\left\{\nabla {\hat{L}}_{h}\left(\beta \right)-\nabla {\hat{L}}_{h}\left(\beta^{*}\right)\right\}\right|}_{\infty }≲\frac{s^{1/2}r{\left(\;\log\;p\right)}^{1/2}}{hN^{1/2}}+\frac{ru^{1/2}}{N^{1/2}}+\frac{s^{1/2}ru}{hN} \end{array}$$

*for any u>0 with probability at least $1-pe^{-u}$.* **Lemma** **A2.** *Let Assumptions* 1 *and* 3 *hold. Then, it holds that*

$$\begin{array}{c} |\nabla {\hat{L}}_{h}\left(\beta^{*}\right){|}_{\infty }≲\frac{{\left(\;\log\;p+u\right)}^{1/2}}{N^{1/2}} \end{array}$$

*for any u>0 with probability at least $1-e^{-u}$.* **Lemma** **A3.** *Let Assumptions* 1–3 *hold. For r>0, l>0 and κ=7/8, it holds with probability at least $1-2p^{-1}$ that*

$$\begin{array}{c} P\left\{A\left(r,l,\kappa \right)\right\}\geq 1-p^{-1} \end{array}$$

*provided that $h\leq {\left(2k_{4}k_{5}\right)}^{-1}$, $r\leq {\left(256k_{2}\right)}^{-1/2}h$ and*

$$\begin{array}{c} n\geq \max\left\{\frac{{\left(55k_{1}k_{5}l\right)}^{2}\;\log\;p}{r^{2}},\frac{18h^{2}\;\log\;p}{r^{2}},\frac{40k_{5}h\;\log\;p}{7}\right\}\;. \end{array}$$

Notice that

(A1) $$\begin{array}{cc} \nabla {\hat{L}}_{h}\left(\beta^{*}\right)= & \;\nabla {\hat{L}}_{1,h}\left(\beta^{*}\right)-\nabla {\hat{L}}_{1,h}\left(\beta^{\left(0\right)}\right)+\nabla {\hat{L}}_{h}\left(\beta^{\left(0\right)}\right)\\ = & \;\left(1-E\right)\{\nabla {\hat{L}}_{1,h}\left(\beta^{*}\right)-\nabla {\hat{L}}_{1,h}\left(\beta^{\left(0\right)}\right)\}\\ \; & +\left(1-E\right)\left\{\nabla {\hat{L}}_{h}\left(\beta^{\left(0\right)}\right)-\nabla {\hat{L}}_{h}\left(\beta^{*}\right)\right\}+\nabla {\hat{L}}_{h}\left(\beta^{*}\right)\;. \end{array}$$

Let $\mu ={\hat{\beta }}^{\left(1\right)}-\beta^{*}$. As we will show in Appendix A.1, Appendix A.2 and Appendix A.3,

(A2) $$\begin{array}{c} \langle \nabla {\hat{L}}_{h}\left(\beta^{*}+\mu \right)-\nabla {\hat{L}}_{h}\left(\beta^{*}\right),\mu \rangle \geq \frac{{7|\mu |}_{\Sigma }^{2}}{8}-\frac{\tau_{1}\;\log\;p{\left|\mu \right|}_{1}^{2}}{n}\;,\;\;\forall {\left|\mu \right|}_{\Sigma }\leq r_{1}\;, \end{array}$$

(A3) $$\begin{array}{c} \langle \nabla {\hat{L}}_{h}\left(\beta^{*}+\mu \right)-{\hat{L}}_{h}\left(\beta^{*}\right),\mu \rangle \geq \frac{7r_{1}{\left|\mu \right|}_{\Sigma }}{8}-\frac{2R\tau_{1}\;\log\;p{\left|\mu \right|}_{1}}{n}\;,\;\;\forall {\left|\mu \right|}_{\Sigma }>r_{1}\;, \end{array}$$

provided that $16k_{2}^{1/2}r_{1}\leq h\leq {\left(2k_{4}k_{5}\right)}^{-1}$, $\tau_{1}r_{1}^{2}\geq 7{\left(55k_{1}k_{5}\right)}^{2}/8$ and ${\left(18h^{2}\;\log\;p/n\right)}^{1/2}\leq r_{1}\leq {\left(32k_{2}^{1/2}k_{4}k_{5}\right)}^{-1}$, with probability at least $1-p^{-1}$, where $\tau_{1}>0$ and $r_{1}>0$. And

(A4) $$\begin{array}{c} {\left|\mu \right|}_{\Sigma }\leq r_{1} \end{array}$$

with probability at least $1-p^{-1}$, provided that $16k_{2}^{1/2}r_{1}\leq h\leq {\left(2k_{4}k_{5}\right)}^{-1}$, $\tau_{1}r_{1}^{2}\geq 7{\left(55k_{1}k_{5}\right)}^{2}/8$ and ${\left(18h^{2}\;\log\;p/n\right)}^{1/2}\leq r_{1}\leq {\left(32k_{2}^{1/2}k_{4}k_{5}\right)}^{-1}$ and

(A5) $$\begin{array}{c} R\leq \min\left\{\frac{7r_{1}^{2}}{4k_{8}\lambda_{1}},{\left(\frac{7r_{1}^{2}n}{8\tau_{1}\;\log\;p}\right)}^{1/2},{\tilde{C}}_{1}r_{1}^{2}\left(\frac{n^{1/2}}{r_{0}{\left(s\;\log\;p\right)}^{1/2}}+\frac{N^{1/2}}{{\left(\;\log\;p\right)}^{1/2}}\right)\right\}\;, \end{array}$$

where ${\tilde{C}}_{1}>0$ is sufficiently small constant. Then, by (A2) and (A4), we have

(A6) $$\begin{array}{c} \langle \nabla {\hat{L}}_{h}\left({\hat{\beta }}^{\left(1\right)}\right)-\nabla {\hat{L}}_{h}\left(\beta^{*}\right),\mu \rangle \geq \frac{{7|\mu |}_{\Sigma }^{2}}{8}-\frac{\tau_{1}\;\log\;p{\left|\mu \right|}_{1}^{2}}{n}\;. \end{array}$$

with probability at least $1-2p^{-1}$, provided that $16k_{2}^{1/2}r_{1}\leq h\leq {\left(2k_{4}k_{5}\right)}^{-1}$, $\tau_{1}r_{1}^{2}\geq 7{\left(55k_{1}k_{5}\right)}^{2}/8$ and ${\left(18h^{2}\;\log\;p/n\right)}^{1/2}\leq r_{1}\leq {\left(32k_{2}^{1/2}k_{4}k_{5}\right)}^{-1}$ and *R* satisfies (A5). Recall $p_{\lambda_{1},\mu }\left(y\right)=p_{\lambda_{1}}\left(y\right)+\mu y^{2}/2$. By Assumption 4(v), we have

$$\begin{array}{c} p_{\lambda_{1},\mu }\left(\beta^{*}\right)-p_{\lambda_{1},\mu }\left({\hat{\beta }}^{\left(1\right)}\right)\geq \langle \nabla p_{\lambda_{1},\mu }\left({\hat{\beta }}^{\left(1\right)}\right),\beta^{*}-{\hat{\beta }}^{\left(1\right)}\rangle =\langle \nabla p_{\lambda_{1}}\left({\hat{\beta }}^{\left(1\right)}\right)+\mu {\hat{\beta }}^{\left(1\right)},\beta^{*}-{\hat{\beta }}^{\left(1\right)}\rangle \;, \end{array}$$

which implies that

$$\begin{array}{c} \langle \nabla p_{\lambda_{1}}\left({\hat{\beta }}^{\left(1\right)}\right),\beta^{*}-{\hat{\beta }}^{\left(1\right)}\rangle \leq p_{\lambda_{1}}\left(\beta^{*}\right)-p_{\lambda_{1}}\left({\hat{\beta }}^{\left(1\right)}\right)+\frac{\mu }{2}\cdot {\left|{\hat{\beta }}^{\left(1\right)}-\beta^{*}\right|}_{2}^{2}\;. \end{array}$$

Together with (A6) and the fact that $\langle \nabla {\hat{L}}_{h}\left({\hat{\beta }}^{\left(1\right)}\right)+\nabla p_{\lambda_{1}}\left({\hat{\beta }}^{\left(1\right)}\right),\beta^{*}-{\hat{\beta }}^{\left(1\right)}\rangle \geq 0$, we have

$$\begin{array}{c} \frac{{7|\mu |}_{\Sigma }^{2}}{8}-\frac{\tau_{1}\;\log\;p{\left|\mu \right|}_{1}^{2}}{n}\leq -\langle \nabla {\hat{L}}_{h}\left(\beta^{*}\right),\mu \rangle +p_{\lambda_{1}}\left(\beta^{*}\right)-p_{\lambda_{1}}\left({\hat{\beta }}^{\left(1\right)}\right)+\frac{\mu }{2}\cdot {\left|\mu \right|}_{2}^{2}\;. \end{array}$$

with probability at least $1-3p^{-1}$, provided that $16k_{2}^{1/2}r_{1}\leq h\leq {\left(2k_{4}k_{5}\right)}^{-1}$, $\tau_{1}r_{1}^{2}\geq 7{\left(55k_{1}k_{5}\right)}^{2}/8$ and ${\left(18h^{2}\;\log\;p/n\right)}^{1/2}\leq r_{1}\leq {\left(32k_{2}^{1/2}k_{4}k_{5}\right)}^{-1}$ and *R* satisfies (A5). Write

(A7) $$\begin{array}{c} \Pi_{1}\left(r\right)=\underset{\beta \in B_{\Sigma }\left(\beta^{*},r\right)\cap \Lambda }{\sup}{\left|\left(1-E\right)\left\{\nabla {\hat{L}}_{h}\left(\beta \right)-\nabla {\hat{L}}_{h}\left(\beta^{*}\right)\right\}\right|}_{\infty }\;, \end{array}$$

(A8) $$\begin{array}{c} \Pi_{2}\left(r\right)=\underset{\beta \in B_{\Sigma }\left(\beta^{*},r\right)\cap \Lambda }{\sup}{\left|\left(1-E\right)\left\{\nabla {\hat{L}}_{1,h}\left(\beta \right)-\nabla {\hat{L}}_{1,h}\left(\beta^{*}\right)\right\}\right|}_{\infty }\;, \end{array}$$

(A9) $$\begin{array}{c} \Pi_{3}={\left|\nabla {\hat{L}}_{h}\left(\beta^{*}\right)\right|}_{\infty }\;. \end{array}$$

Recall ${\left|\mu \right|}_{1}\leq 2R$. Due to ${\left|\mu \right|}_{2}^{2}\leq k_{3}{\left|\mu \right|}_{\Sigma }^{2}$, by (A1), it holds that

(A10) $$\begin{array}{cc} \frac{7-4k_{3}\mu }{8}\cdot {\left|\mu \right|}_{\Sigma }^{2}\leq & \;p_{\lambda_{1}}\left(\beta^{*}\right)-p_{\lambda_{1}}\left({\hat{\beta }}^{\left(1\right)}\right)-\langle \nabla {\hat{L}}_{h}\left(\beta^{*}\right),\mu \rangle +\frac{\tau_{1}\;\log\;p{\left|\mu \right|}_{1}^{2}}{n}\\ \leq & \;p_{\lambda_{1}}\left(\beta^{*}\right)-p_{\lambda_{1}}\left({\hat{\beta }}^{\left(1\right)}\right)+\left\{\Pi_{1}\left(r_{0}\right)+\Pi_{2}\left(r_{0}\right)+\Pi_{3}\right\}{\left|\mu \right|}_{1}+\frac{\tau_{1}\;\log\;p{\left|\mu \right|}_{1}^{2}}{n}\\ \leq & \;p_{\lambda_{1}}\left(\beta^{*}\right)-p_{\lambda_{1}}\left({\hat{\beta }}^{\left(1\right)}\right)+\left\{\Pi_{1}\left(r_{0}\right)+\Pi_{2}\left(r_{0}\right)+\Pi_{3}+\frac{2R\tau_{1}\;\log\;p}{n}\right\}\cdot {\left|\mu \right|}_{1}\;, \end{array}$$

with probability at least $1-2p^{-1}$, provided that $16k_{2}^{1/2}r_{1}\leq h\leq {\left(2k_{4}k_{5}\right)}^{-1}$, $\tau_{1}r_{1}^{2}\geq 7{\left(55k_{1}k_{5}\right)}^{2}/8$ and ${\left(18h^{2}\;\log\;p/n\right)}^{1/2}\leq r_{1}\leq {\left(32k_{2}^{1/2}k_{4}k_{5}\right)}^{-1}$ and *R* satisfies (A5). Let $\lambda_{1}$ satisfy that

$$\begin{array}{c} \lambda_{1}\geq 4k_{8}^{-1}\left\{\Pi_{1}\left(r_{0}\right)+\Pi_{2}\left(r_{0}\right)+\Pi_{3}+\frac{2R\tau_{1}\;\log\;p}{n}\right\}\;. \end{array}$$

Recall $0<\mu <7/(5k_{3})$. Then, by Equation (53) of Loh and Wainwright [21], we have

$$\begin{array}{cc} \frac{7-4k_{3}\mu }{8}\cdot {\left|\mu \right|}_{\Sigma }^{2}\leq & \;p_{\lambda_{1}}\left(\beta^{*}\right)-p_{\lambda_{1}}\left({\hat{\beta }}^{\left(1\right)}\right)+\frac{\lambda_{1}k_{8}{\left|\mu \right|}_{1}}{4}\\ \leq & \;p_{\lambda_{1}}\left(\beta^{*}\right)-p_{\lambda_{1}}\left({\hat{\beta }}^{\left(1\right)}\right)+\frac{p_{\lambda_{1}}\left(\mu \right)}{4}+\frac{{\mu |\mu |}_{2}^{2}}{8}\\ \leq & \;p_{\lambda_{1}}\left(\beta^{*}\right)-p_{\lambda_{1}}\left({\hat{\beta }}^{\left(1\right)}\right)+\frac{p_{\lambda_{1}}\left(\beta^{\left(1\right)}\right)+p_{\lambda_{1}}\left(\beta^{*}\right)}{2}+\frac{k_{3}\mu {\left|\mu \right|}_{\Sigma }^{2}}{8}\\ \leq & \;\frac{3p_{\lambda_{1}}\left(\beta^{*}\right)}{2}-\frac{p_{\lambda_{1}}\left({\hat{\beta }}^{\left(1\right)}\right)}{2}+\frac{k_{3}\mu {\left|\mu \right|}_{\Sigma }^{2}}{8} \end{array}$$

which implies that

(A11) $$\begin{array}{c} 0\leq \frac{7-5k_{3}\mu }{8}\cdot {\left|\mu \right|}_{\Sigma }^{2}\leq \frac{3p_{\lambda_{1}}\left(\beta^{*}\right)}{2}-\frac{p_{\lambda_{1}}\left({\hat{\beta }}^{\left(1\right)}\right)}{2} \end{array}$$

with probability at least $1-2p^{-1}$, provided that $16k_{2}^{1/2}r_{1}\leq h\leq {\left(2k_{4}k_{5}\right)}^{-1}$, $\tau_{1}r_{1}^{2}\geq 7{\left(55k_{1}k_{5}\right)}^{2}/8$ and ${\left(18h^{2}\;\log\;p/n\right)}^{1/2}\leq r_{1}\leq {\left(32k_{2}^{1/2}k_{4}k_{5}\right)}^{-1}$ and *R* satisfies (A5). Let $S=\mathrm{supp}\left(\beta^{*}\right)\subseteq \left[p\right]$ be the true active set with cardinality |S|≤s. Then, by Lemma A.5 of Loh and Wainwright [21], we have

(A12) $$\begin{array}{c} 0\leq 3p_{\lambda_{1}}\left(\beta^{*}\right)-p_{\lambda_{1}}\left({\hat{\beta }}^{\left(1\right)}\right)\leq 3k_{8}\lambda_{1}|\mu_{S}{|}_{1}-k_{8}\lambda_{1}{\left|\mu_{S^{c}}\right|}_{1}\;, \end{array}$$

which implies that $|\mu_{S^{c}}{|}_{1}\leq 3{\left|\mu_{S}\right|}_{1}$, with probability at least $1-2p^{-1}$, provided that $16k_{2}^{1/2}r_{1}\leq h\leq {\left(2k_{4}k_{5}\right)}^{-1}$, $\tau_{1}r_{1}^{2}\geq 7{\left(55k_{1}k_{5}\right)}^{2}/8$ and ${\left(18h^{2}\;\log\;p/n\right)}^{1/2}\leq r_{1}\leq {\left(32k_{2}^{1/2}k_{4}k_{5}\right)}^{-1}$ and *R* satisfies (A5). Hence,

$$\begin{array}{cc} {\left|\mu \right|}_{1}=|\mu_{S}{|}_{1}+{\left|\mu_{S^{c}}\right|}_{1}\leq & \;4|\mu_{S}{|}_{1}\leq 4s^{1/2}{\left|\mu_{S}\right|}_{2}\;, \end{array}$$

with probability at least $1-2p^{-1}$, provided that $16k_{2}^{1/2}r_{1}\leq h\leq {\left(2k_{4}k_{5}\right)}^{-1}$, $\tau_{1}r_{1}^{2}\geq 7{\left(55k_{1}k_{5}\right)}^{2}/8$ and ${\left(18h^{2}\;\log\;p/n\right)}^{1/2}\leq r_{1}\leq {\left(32k_{2}^{1/2}k_{4}k_{5}\right)}^{-1}$ and *R* satisfies (A5). Hence, $\left\{{\hat{\beta }}^{\left(1\right)}\in \Lambda \right\}$, with probability at least $1-2p^{-1}$, provided that $16k_{2}^{1/2}r_{1}\leq h\leq {\left(2k_{4}k_{5}\right)}^{-1}$, $\tau_{1}r_{1}^{2}\geq 7{\left(55k_{1}k_{5}\right)}^{2}/8$ and ${\left(18h^{2}\;\log\;p/n\right)}^{1/2}\leq r_{1}\leq {\left(32k_{2}^{1/2}k_{4}k_{5}\right)}^{-1}$ and *R* satisfies (A5). Then, by (A11) and (A12), we have

$$\begin{array}{cc} \frac{7-5k_{3}\mu }{8}\cdot {\left|\mu \right|}_{\Sigma }^{2}\leq & \;\frac{k_{8}\lambda_{1}\left(3\right|\mu_{S}{|}_{1}-|\mu_{S^{c}}{|}_{1})}{2}\leq \frac{3k_{8}\lambda_{1}{\left|\mu_{S}\right|}_{1}}{2}\leq \frac{3k_{8}{\left(k_{3}s\right)}^{1/2}\lambda_{1}{\left|\mu \right|}_{\Sigma }}{2}\;, \end{array}$$

which implies that

(A13) $$\begin{array}{c} {\left|\mu \right|}_{\Sigma }\leq \frac{3k_{8}{\left(k_{3}s\right)}^{1/2}\lambda_{1}}{2}\;, \end{array}$$

with probability at least $1-2p^{-1}$, provided that $16k_{2}^{1/2}r_{1}\leq h\leq {\left(2k_{4}k_{5}\right)}^{-1}$, $\tau_{1}r_{1}^{2}\geq 7{\left(55k_{1}k_{5}\right)}^{2}/8$ and ${\left(18h^{2}\;\log\;p/n\right)}^{1/2}\leq r_{1}\leq {\left(32k_{2}^{1/2}k_{4}k_{5}\right)}^{-1}$ and *R* satisfies (A5). It remains to choose a sufficiently large $\lambda_{1}$ and *R*. By Lemmas A1 and A2, we have

(A14) $$\begin{array}{cc} \Pi_{1}\left(r_{0}\right)+\Pi_{2}\left(r_{0}\right)+\Pi_{3}≲ & \;s^{1/2}r_{0}\left(\frac{{\left(\;\log\;p\right)}^{1/2}}{bN^{1/2}}+\frac{{\left(\;\log\;p\right)}^{1/2}}{bn^{1/2}}\right)+\frac{{\left(\;\log\;p\right)}^{1/2}}{N^{1/2}}\\ ≲ & \;\frac{r_{0}{\left(s\;\log\;p\right)}^{1/2}}{n^{1/2}}+\frac{{\left(\;\log\;p\right)}^{1/2}}{N^{1/2}} \end{array}$$

with probability at least $1-p^{-1}$. To ensure that $|{\hat{\beta }}^{\left(1\right)}-\beta^{*}{|}_{\Sigma }$ converges as fast as possible, selecting

$$\begin{array}{c} \lambda_{1}≍\frac{r_{0}{\left(s\;\log\;p\right)}^{1/2}}{n^{1/2}}+\frac{{\left(\;\log\;p\right)}^{1/2}}{N^{1/2}}+\frac{R\tau_{1}\;\log\;p}{n}\;. \end{array}$$

By (A5) and (A13), we select $r_{1}=O\left(1\right)$, $\tau_{1}=O\left(1\right)$ and h=O(1), $R≍n^{1/2}{\left(\;\log\;p\right)}^{-1/2}$ and then

(A15) $$\begin{array}{c} \lambda_{1}≍\frac{r_{0}{\left(s\;\log\;p\right)}^{1/2}}{n^{1/2}}+\frac{{\left(\;\log\;p\right)}^{1/2}}{N^{1/2}}\;. \end{array}$$

Together with (A13), it holds that

$$\begin{array}{c} |{\hat{\beta }}^{\left(1\right)}-\beta^{*}{|}_{\Sigma }≲\frac{r_{0}s{\left(\;\log\;p\right)}^{1/2}}{n^{1/2}}+\frac{{\left(s\;\log\;p\right)}^{1/2}}{N^{1/2}}\;, \end{array}$$

with probability at least $1-3p^{-1}$, provided that h=O(1), $R≍n^{1/2}{\left(\;\log\;p\right)}^{-1/2}$ and $\lambda_{1}$ satisfies (A15). Hence, we complete the proof of Theorem 1. □

### Appendix A.1. Proof of (A2)

**Proof** **of** **(A2).** Case 1: ${\left|\mu \right|}_{1}/{\left|\mu \right|}_{\Sigma }\geq {\left\{7n/\left(8\tau_{1}\;\log\;p\right)\right\}}^{1/2}$. Due to $\hat{L}\left(\cdot \right)$ being convex, it holds that

(A16) $$\begin{array}{c} \langle \nabla {\hat{L}}_{h}\left(\beta^{*}+\mu \right)-\nabla {\hat{L}}_{h}\left(\beta^{*}\right),\mu \rangle \geq 0\geq \frac{{7|\mu |}_{\Sigma }^{2}}{8}-\frac{\tau_{1}\;\log\;p{\left|\mu \right|}_{1}^{2}}{n}\;. \end{array}$$

Case 2: ${\left|\mu \right|}_{1}/{\left|\mu \right|}_{\Sigma }<{\left\{7n/\left(8\tau_{1}\;\log\;p\right)\right\}}^{1/2}$. Recall n≫logp. For any ${\left|\mu \right|}_{\Sigma }\leq r_{1}$, by Assumption 1, we have ${\left|\mu \right|}_{\Sigma }\leq k_{3}^{1/2}$. By Lemma A3 with $l={\left\{7n/\left(8\tau_{1}\;\log\;p\right)\right\}}^{1/2}$, we have

(A17) $$\begin{array}{c} \langle \nabla {\hat{L}}_{h}\left(\beta^{*}+\mu \right)-\nabla {\hat{L}}_{h}\left(\beta^{*}\right),\mu \rangle \geq \frac{{7|\mu |}_{\Sigma }^{2}}{8} \end{array}$$

provided that $16k_{2}^{1/2}r_{1}\leq h\leq {\left(2k_{4}k_{5}\right)}^{-1}$, $\tau_{1}r_{1}^{2}\geq 7{\left(55k_{1}k_{5}\right)}^{2}/8$ and ${\left(18h^{2}\;\log\;p/n\right)}^{1/2}\leq r_{1}\leq {\left(32k_{2}^{1/2}k_{4}k_{5}\right)}^{-1}$, with probability at least $1-p^{-1}$. Combining (A16) and (A17), (A2) holds. □

### Appendix A.2. Proof of (A3)

**Proof of** **(A3).** Let $f\left(t\right)={\hat{L}}_{h}\left(\beta^{*}+t\mu \right)$. Since ${\hat{L}}_{h}\left(\cdot \right)$ is convex, f(t) is also convex, and $f^{\prime }\left(1\right)-f^{\prime }\left(0\right)\geq f^{\prime }\left(t\right)-f^{\prime }\left(0\right)$ for t∈[0,1]. Then, we obtain that

$$\begin{array}{c} \langle \nabla {\hat{L}}_{h}\left(\beta^{*}+\mu \right)-\nabla {\hat{L}}_{h}\left(\beta^{*}\right),\mu \rangle \geq \frac{1}{t}\times \langle \nabla {\hat{L}}_{h}\left(\beta^{*}+t\mu \right)-\nabla {\hat{L}}_{h}\left(\beta^{*}\right),t\mu \rangle \;. \end{array}$$

Selecting $t=r_{1}/{\left|\mu \right|}_{\Sigma }\in \left(0,1\right)$, by (A2), we have

$$\begin{array}{cc} \langle \nabla {\hat{L}}_{h}\left(\beta^{*}+\mu \right)-\nabla {\hat{L}}_{h}\left(\beta^{*}\right),\mu \rangle \geq & \;\frac{{\left|\mu \right|}_{\Sigma }}{r_{1}}\left(\frac{7}{8}|\frac{\mu }{{\left|\mu \right|}_{\Sigma }}{|}_{\Sigma }^{2}-\frac{\tau_{1}r_{1}^{2}\;\log\;p}{n}\times |\frac{\mu }{{\left|\mu \right|}_{\Sigma }}{|}_{1}^{2}\right)\\ = & \;\frac{{\left|\mu \right|}_{\Sigma }}{r_{1}}\left(\frac{7r_{1}^{2}}{8}-\frac{\tau_{1}r_{1}^{2}\;\log\;p}{n}\times \frac{{\left|\mu \right|}_{1}^{2}}{{\left|\mu \right|}_{\Sigma }^{2}}\right)\\ \geq & \;\frac{{\left|\mu \right|}_{\Sigma }}{r_{1}}\left(\frac{7r_{1}^{2}}{8}-\frac{2R\tau_{1}r_{1}^{2}\;\log\;p}{n}\times \frac{{\left|\mu \right|}_{1}}{{\left|\mu \right|}_{\Sigma }^{2}}\right)\\ \geq & \;\frac{{\left|\mu \right|}_{\Sigma }}{r_{1}}\left(\frac{7r_{1}^{2}}{8}-\frac{2R\tau_{1}r_{1}\;\log\;p}{n}\times \frac{{\left|\mu \right|}_{1}}{{\left|\mu \right|}_{\Sigma }}\right)\\ = & \;\frac{7r_{1}{\left|\mu \right|}_{\Sigma }}{8}-\frac{2R\tau_{1}\;\log\;p{\left|\mu \right|}_{1}}{n} \end{array}$$

provided that $16k_{2}^{1/2}r_{1}\leq h\leq {\left(2k_{4}k_{5}\right)}^{-1}$, $\tau_{1}r_{1}^{2}\geq 7{\left(55k_{1}k_{5}\right)}^{2}/8$ and ${\left(18h^{2}\;\log\;p/n\right)}^{1/2}\leq r_{1}\leq {\left(32k_{2}^{1/2}k_{4}k_{5}\right)}^{-1}$, with probability at least $1-p^{-1}$. Hence, (A3) holds. □

### Appendix A.3. Proof of (A4)

**Proof** **of** **(A4).** If ${\left|\mu \right|}_{\Sigma }>r_{1}$, by $\nabla {\hat{L}}_{h}\left(\beta^{*}+\mu \right)=-\nabla p_{\lambda_{1}}\left({\hat{\beta }}^{\left(1\right)}\right)$ and (A3), we have

(A18) $$\begin{array}{c} \langle -\nabla p_{\lambda_{1}}\left({\hat{\beta }}^{\left(1\right)}\right)-\nabla {\hat{L}}_{h}\left(\beta^{*}\right),\mu \rangle \geq \frac{7r_{1}{\left|\mu \right|}_{\Sigma }}{8}-\frac{2R\tau_{1}\;\log\;p{\left|\mu \right|}_{1}}{n} \end{array}$$

provided that $16k_{2}^{1/2}r_{1}\leq h\leq {\left(2k_{4}k_{5}\right)}^{-1}$, $\tau_{1}r_{1}^{2}\geq 7{\left(55k_{1}k_{5}\right)}^{2}/8$ and ${\left(18h^{2}\;\log\;p/n\right)}^{1/2}\geq r_{1}\geq {\left(32k_{2}^{1/2}k_{4}k_{5}\right)}^{-1}$, with probability at least $1-2p^{-1}$. Moreover by Assumption 4, the Hölder inequality and Lemma 4 of Loh and Wainwright [21], restricted on $E_{0}\left(r_{0}\right)$, we have

(A19) $$\begin{array}{cc} \; & \langle -\nabla p_{\lambda_{1}}\left({\hat{\beta }}^{\left(1\right)}\right)-\nabla {\hat{L}}_{h}\left(\beta^{*}\right),\mu \rangle \\ \; & \;\;\;\leq [|\nabla p_{\lambda_{1}}\left({\hat{\beta }}^{\left(1\right)}\right){|}_{\infty }+\underset{\beta -\beta^{*}\in B_{\Sigma }\left(\beta^{*},r_{0}\right)\cap \Lambda }{\sup}{\left|\left(1-E\right)\left\{\nabla {\hat{L}}_{h}\left(\beta \right)-\nabla {\hat{L}}_{h}\left(\beta^{*}\right)\right\}\right|}_{\infty }\\ \; & \;\;\;\;\;\;\;\;\;\;\;+\underset{\beta \in B_{\Sigma }\left(\beta^{*},r_{0}\right)\cap \Lambda }{\sup}|\left(1-E\right)\left\{\nabla {\hat{L}}_{1,h}\left(\beta \right)-\nabla {\hat{L}}_{1,h}\left(\beta^{*}\right)\right\}{|}_{\infty }+|\nabla {\hat{L}}_{h}\left(\beta^{*}\right){|}_{\infty }]\cdot {\left|\mu \right|}_{1}\\ \; & \;\;\;\leq k_{8}\lambda_{1}{\left|\mu \right|}_{1}+\left\{\Pi_{1}\left(r_{0}\right)+\Pi_{2}\left(r_{0}\right)+\Pi_{3}\right\}{\left|\mu \right|}_{1}\;. \end{array}$$

Recall ${\left|\mu \right|}_{1}\leq 2R$. Together with (A18), selecting u=2logp, by Lemmas A1 and A2, we have

$$\begin{array}{cc} \frac{7r_{1}{\left|\mu \right|}_{\Sigma }}{8}\leq & \;\left(k_{8}\lambda_{1}+\frac{2R\tau_{1}\;\log\;p}{n}\right){\left|\mu \right|}_{1}+\left\{\Pi_{1}\left(r_{0}\right)+\Pi_{2}\left(r_{0}\right)+\Pi_{3}\right\}{\left|\mu \right|}_{1}\\ \leq & \;2\left(k_{8}\lambda_{1}+\frac{2R\tau_{1}\;\log\;p}{n}\right)R+2\left\{\Pi_{1}\left(r_{0}\right)+\Pi_{2}\left(r_{0}\right)+\Pi_{3}\right\}R\\ \leq & \;2\left(k_{8}\lambda_{1}+\frac{2R\tau_{1}\;\log\;p}{n}\right)R+C_{1}\left\{s^{1/2}r_{0}\left(\frac{{\left(\;\log\;p\right)}^{1/2}}{bN^{1/2}}+\frac{{\left(\;\log\;p\right)}^{1/2}}{bn^{1/2}}\right)+\frac{{\left(\;\log\;p\right)}^{1/2}}{N^{1/2}}\right\}R\\ \leq & \;2\left(k_{8}\lambda_{1}+\frac{2R\tau_{1}\;\log\;p}{n}\right)R+C_{2}\left\{\frac{r_{0}s^{1/2}{\left(\;\log\;p\right)}^{1/2}}{n^{1/2}}+\frac{{\left(\;\log\;p\right)}^{1/2}}{N^{1/2}}\right\}R \end{array}$$

which implies that

$$\begin{array}{c} {\left|\mu \right|}_{\Sigma }\leq \frac{16}{7r_{1}}\left(k_{8}\lambda_{1}+\frac{2R\tau_{1}\;\log\;p}{n}\right)R+\frac{C_{3}}{r_{1}}\left\{\frac{r_{0}s^{1/2}{\left(\;\log\;p\right)}^{1/2}}{n^{1/2}}+\frac{{\left(\;\log\;p\right)}^{1/2}}{N^{1/2}}\right\}R\leq r_{1} \end{array}$$

provided that $16k_{2}^{1/2}r_{1}\leq h\leq {\left(2k_{4}k_{5}\right)}^{-1}$, $\tau_{1}r_{1}^{2}\geq 7{\left(55k_{1}k_{5}\right)}^{2}/8$ and ${\left(18h^{2}\;\log\;p/n\right)}^{1/2}\geq r_{1}\leq {\left(32k_{2}^{1/2}k_{4}k_{5}\right)}^{-1}$ and

$$\begin{array}{c} R\leq \min\left\{\frac{7r_{1}^{2}}{4k_{8}\lambda_{1}},{\left(\frac{7r_{1}^{2}n}{8\tau_{1}\;\log\;p}\right)}^{1/2},{\tilde{C}}_{1}r_{1}^{2}\left(\frac{n^{1/2}}{r_{0}{\left(s\;\log\;p\right)}^{1/2}}+\frac{N^{1/2}}{{\left(\;\log\;p\right)}^{1/2}}\right)\right\}\;, \end{array}$$

where ${\tilde{C}}_{1}>0$ is a sufficiently small constant. Hence, (A4) holds. □

## Appendix B. Proof of Theorem 2

**Proof of** **Theorem 2.** To proof Theorem 2, we need the following lemma, whose proof is given in Appendix C.6. **Lemma** **A4.** *Let Assumptions* 1–4 *hold. Let h=O(1), $R≍{\left(n/\;\log\;p\right)}^{1/2}$ and $\lambda_{0}≍{\left(\;\log\;p/n\right)}^{1/2}$. Then, it holds with probability at least $1-3p^{-1}$ that $\left\{{\hat{\beta }}^{\left(0\right)}\in \Lambda \right\}$ and*

$$\begin{array}{c} |{\hat{\beta }}^{\left(0\right)}-\beta^{*}{|}_{\Sigma }≲\frac{{\left(s\;\log\;p\right)}^{1/2}}{n^{1/2}}\;. \end{array}$$

By Theorem 1, ${\hat{\beta }}^{\left(1\right)}$ satisfies

$$\begin{array}{c} |{\hat{\beta }}^{\left(1\right)}-\beta^{*}{|}_{\Sigma }\leq \frac{C_{1}r_{0}s{\left(\;\log\;p\right)}^{1/2}}{n^{1/2}}+\frac{C_{2}{\left(s\;\log\;p\right)}^{1/2}}{N^{1/2}} \end{array}$$

and $\left\{{\hat{\beta }}^{\left(1\right)}\in \Lambda \right\}$, for $\lambda_{1}≍r_{0}{\left(s\;\log\;p/n\right)}^{1/2}+{\left(\;\log\;p/N\right)}^{1/2}$ and $R≍{\left(n/\;\log\;p\right)}^{1/2}$, with probability at least $1-3p^{-1}$. Write

$$\begin{array}{c} \vartheta =\frac{C_{1}s{\left(\;\log\;p\right)}^{1/2}}{n^{1/2}}\;. \end{array}$$

We have ϑ<1, provided that $n\gg s^{2}\;\log\;p$. For t∈[T]/{1}, define the event $E_{t}\left(t_{t}\right)=\left\{{\hat{\beta }}^{\left(t\right)}\in B_{\Sigma }\left(\beta^{*},r_{t}\right)\cap \Lambda \right\}$ with

$$\begin{array}{c} r_{t}=\vartheta r_{t-1}+\frac{C_{2}{\left(s\;\log\;p\right)}^{1/2}}{N^{1/2}}=\vartheta^{t}r_{0}+\frac{C_{2}\left(1-\vartheta^{t}\right)}{1-\vartheta }\times \frac{{\left(s\;\log\;p\right)}^{1/2}}{N^{1/2}}\;. \end{array}$$

At iteration t∈[T]/{1}, let

$$\begin{array}{c} \lambda_{t}≍\frac{{\left(\;\log\;p\right)}^{1/2}}{N^{1/2}}+\vartheta r_{t-1}≍\frac{{\left(\;\log\;p\right)}^{1/2}}{N^{1/2}}+\vartheta^{t}r_{0}\;. \end{array}$$

Applying Theorem 1 repeatedly, we obtain that conditioned on the event $E(t_{t-1})$, the *t*-th iterate ${\hat{\beta }}^{\left(t\right)}$ satisfies ${\hat{\beta }}^{\left(t\right)}\in \Lambda$ and

(A20) $$\begin{array}{c} |{\hat{\beta }}^{\left(t\right)}-\beta^{*}{|}_{\Sigma }\leq r_{t}=\vartheta^{t}r_{0}+\frac{C_{2}\left(1-\vartheta^{t}\right)}{1-\vartheta }\times \frac{{\left(s\;\log\;p\right)}^{1/2}}{N^{1/2}} \end{array}$$

with probability at least $1-3p^{-1}$, provided that h=O(1) and $R≍{\left(n/\;\log\;p\right)}^{1/2}$. Selecting $r_{0}≍{\left(s\;\log\;p/n\right)}^{1/2}$, by Lemma A4, it holds that

$$\begin{array}{cc} \; & P\left\{|{\hat{\beta }}^{\left(t\right)}-\beta^{*}{|}_{\Sigma }\leq \frac{C_{3}s^{(2t+1)/2}{\left(\;\log\;p\right)}^{(t+1)/2}}{n^{(t+1)/2}}+\frac{{\left(s\;\log\;p\right)}^{1/2}}{N^{1/2}}\right\}\\ \; & \;\;\;\leq P\left\{|{\hat{\beta }}^{\left(t\right)}-\beta^{*}{|}_{\Sigma }\leq \frac{C_{3}s^{(2t+1)/2}{\left(\;\log\;p\right)}^{(t+1)/2}}{n^{(t+1)/2}}+\frac{{\left(s\;\log\;p\right)}^{1/2}}{N^{1/2}},E\left(r_{0}\right)\right\}+P\left[{\left\{E\left(r_{0}\right)\right\}}^{c}\right]\\ \; & \;\;\;\leq 3Tp^{-1}+3p^{-1}=3\left(T+1\right)p^{-1} \end{array}$$

provided that $n\gg s^{2}\;\log\;p$ and $\lambda_{t}≍{\left(\;\log\;p/N\right)}^{1/2}+s^{(2t+1)/2}{\left(\;\log\;p/n\right)}^{(t+1)/2}$. Hence, we complete the proof of Theorem 2. □

## Appendix C. Proofs of the Auxiliary Lemmas

### Appendix C.1. Proof of Lemma A1

**Proof of** **Lemma A1.** Given $r_{1},r_{2}>0$, define the local cone-neighborhood of $\beta^{*}$

$$\begin{array}{c} \Theta_{0}\left(r_{1},r_{2}\right)=\{\upsilon \in R^{p}{:|\upsilon |}_{\Sigma }\leq r_{1}{,|\upsilon |}_{1}\leq r_{2}\}\;. \end{array}$$

Write $\upsilon =\beta -\beta^{*}$ and

$$\begin{array}{c} f_{k}\left(\xi_{i},\xi_{j},\upsilon \right)=\left\{L_{h}^{\prime }\left(\varepsilon_{i}-\varepsilon_{j}-{\left(X_{i}-X_{j}\right)}^{⊤}\upsilon \right)-L_{h}^{\prime }\left(\varepsilon_{i}-\varepsilon_{j}\right)\right\}\left(X_{i,k}-X_{j,k}\right) \end{array}$$

for k∈[p] and $\xi_{i}=\left(X_{i},\varepsilon_{i}\right)$. Then,

(A21) $$\begin{array}{cc} \; & \underset{\beta -\beta^{*}\in B_{\Sigma }\left(r\right)\cap \Lambda }{\sup}{\left|\left(1-E\right)\left\{\nabla {\hat{L}}_{h}\left(\beta \right)-\nabla {\hat{L}}_{h}\left(\beta^{*}\right)\right\}\right|}_{\infty }\\ \; & \;\;\;\;\;\;\;\;\;\;\;\;\;\;\;\;=\max_{k\in [p]}\underset{\upsilon \in \Theta_{0}\left(r,4s^{1/2}r\right)}{\sup}|\frac{1}{N(N-1)}\sum_{i=1}^{N}\sum_{j\neq i}\left(1-E\right)\left\{f_{k}\left(\xi_{i},\xi_{j},\upsilon \right)\right\}|\;. \end{array}$$

Recall $L_{h}^{\prime }\left(u\right)=2\int_{0}^{u}K_{h}\left(v\right)\;dv$ with $K_{h}\left(v\right)=k\left(v/h\right)/h$. By Assumptions 1 and 3, for any $\upsilon \in \Theta_{0}\left(r,l\right)$, we have

$$\begin{array}{cc} |f_{k}\left(\xi_{i},\xi_{j},\upsilon \right)|\leq & \;C_{1}\max_{i,j\in [n],k\in [d]}|X_{i,k}-X_{j,k}\left|\cdot \right|{\left(X_{i}^{⊤}-X_{j}^{⊤}\right)}^{⊤}\upsilon |/h\\ \leq & \;C_{2}\left(\right|X_{i}{|}_{\infty }+|X_{j}{|}_{\infty }{)\cdot |\upsilon |}_{1}/h\\ \leq & \;C_{3}s^{1/2}r/h\;. \end{array}$$

By Assumption 3 again, for any $\upsilon \in \Theta_{0}\left(r,l\right)$, we have

$$\begin{array}{cc} \; & E\{L_{h}^{\prime }\left(\varepsilon_{i}-\varepsilon_{j}-{\left(X_{i}-X_{j}\right)}^{⊤}\upsilon \right)-L_{h}^{\prime }\left(\varepsilon_{i}-\varepsilon_{j}\right)\;|\;X_{i},X_{j}\}\\ \; & \;\;\;\;\;\;\;\;\;=2E\left\{\int_{\varepsilon_{i}-\varepsilon_{j}}^{\varepsilon_{i}-\varepsilon_{j}-{\left(X_{i}-X_{j}\right)}^{⊤}\upsilon }K_{h}\left(v\right)\;dv\;|\;X_{i},X_{j}\right\}\\ \; & \;\;\;\;\;\;\;\;\;=2E\left\{\int_{0}^{-{\left(X_{i}-X_{j}\right)}^{⊤}\upsilon }K\left(u+\left(\varepsilon_{i}+\varepsilon_{j}\right)/h\right)\;du\;|\;X_{i},X_{j}\right\}\\ \; & \;\;\;\;\;\;\;\;\;\leq C_{1}\left|{\left(X_{i}-X_{j}\right)}^{⊤}\upsilon \right|\;, \end{array}$$

which implies that

$$\begin{array}{cc} E\{f_{k}^{2}\left(\xi_{i},\xi_{j},\upsilon \right)\}\leq & \;C_{1}E\{|{\left(X_{i}-X_{j}\right)}^{⊤}\upsilon {|}^{2}\left(X_{i,k}-X_{j,k}\right)\}\\ \leq & \;C_{1}{\left[E\left\{{\left(X_{i,k}-X_{j,k}\right)}^{4}\right\}\right]}^{1/2}\left[E\{\right|{\left(X_{i}-X_{j}\right)}^{⊤}{\upsilon |}^{4}{\}]}^{1/2}\\ \leq & \;C_{2}{\left[E\left\{{\left(X_{i}^{⊤}\upsilon \right)}^{4}\right\}\right]}^{1/2}+C_{2}{\left[E\left\{{\left(X_{j}^{⊤}\upsilon \right)}^{4}\right\}\right]}^{1/2}≲1\;. \end{array}$$

By Theorem 2 of Rejchel [24], we have

(A22) $$\begin{array}{cc} \; & \underset{\upsilon \in \Theta_{0}\left(r,l\right)}{\sup}|\frac{1}{N(N-1)}\sum_{i=1}^{N}\sum_{j\neq i}\left(1-E\right)\left\{f_{k}\left(\xi_{i},\xi_{j},\upsilon \right)\right\}|\\ \; & \;\;\;\;\;\;≲E\left\{\underset{\upsilon \in \Theta_{0}\left(r,l\right)}{\sup}|\frac{1}{\left\lfloor N/2\right\rfloor }\sum_{i=1}^{\left\lfloor N/2\right\rfloor }\sigma_{i}f_{k}\left(\xi_{i},\xi_{\lfloor N/2\rfloor +i},\upsilon \right)|\right\}+\frac{ru^{1/2}}{N^{1/2}}+\frac{s^{1/2}ru}{hN} \end{array}$$

for any u>0 with probability at least $1-e^{-u}$, where ${\left\{\sigma_{i}\right\}}_{i=1}^{\left\lfloor N/2\right\rfloor }$ is a sequence of Rademacher random variables. For each *i*, write $f_{k}\left(\xi_{i},\xi_{\lfloor N/2\rfloor +i},\upsilon \right)=\Upsilon_{i}\left({\left(X_{i}-X_{\lfloor N/2\rfloor +i}\right)}^{⊤}\upsilon \right)$, where $\Upsilon_{i}\left(\cdot \right)$ is such that $\Upsilon_{i}\left(0\right)=0$ and $|\Upsilon_{i}\left(u\right)-\Upsilon_{i}\left(v\right)|≲h^{-1}|X_{i,k}-X_{\lfloor N/2\rfloor +i}\left|\cdot |u-v\right|$. Then, by the Talagrand contraction principle,

$$\begin{array}{cc} \; & E\left\{\underset{\upsilon \in \Theta_{0}\left(r,l\right)}{\sup}|\frac{1}{\left\lfloor N/2\right\rfloor }\sum_{i=1}^{\left\lfloor N/2\right\rfloor }\sigma_{i}f_{k}\left(\xi_{i},\xi_{\lfloor N/2\rfloor +i},\upsilon \right)|\right\}\\ \; & \;\;\;\leq C_{3}E\left\{\underset{\upsilon \in \Theta_{0}\left(r,4s^{1/2}r\right)}{\sup}|\frac{1}{\left\lfloor N/2\right\rfloor }\sum_{i=1}^{\left\lfloor N/2\right\rfloor }\sigma_{i}h^{-1}\left|X_{i,k}-X_{\lfloor N/2\rfloor +i}\right|\cdot {\left(X_{i}-X_{\lfloor N/2\rfloor +i}\right)}^{⊤}\upsilon |\right\}\\ \; & \;\;\;\leq \frac{C_{4}s^{1/2}r}{h}E\left\{|\frac{1}{\left\lfloor N/2\right\rfloor }\sum_{i=1}^{\left\lfloor N/2\right\rfloor }\sigma_{i}\left|X_{i,k}-X_{\lfloor N/2\rfloor +i}\right|\left(X_{i}-X_{\lfloor N/2\rfloor +i}\right){|}_{\infty }\right\}\\ \; & \;\;\;≲\frac{s^{1/2}r{\left(\;\log\;p\right)}^{1/2}}{hN^{1/2}}\;. \end{array}$$

Together with (A22), by (A21) and Bonferroni inequality, it holds that

$$\begin{array}{c} \underset{\beta -\beta^{*}\in B_{\Sigma }\left(r\right)\cap \Lambda }{\sup}{\left|\left(1-E\right)\left\{\nabla {\hat{L}}_{h}\left(\beta \right)-\nabla {\hat{L}}_{h}\left(\beta^{*}\right)\right\}\right|}_{\infty }≲\frac{s^{1/2}r{\left(\;\log\;p\right)}^{1/2}}{hN^{1/2}}+\frac{ru^{1/2}}{N^{1/2}}+\frac{s^{1/2}ru}{hN} \end{array}$$

for any u>0 with probability at least $1-pe^{-u}$. Hence, we complete the proof of Lemma A1. □

### Appendix C.2. Proof of Lemma A2

**Proof of** **Lemma A2.** Recall $L_{h}^{\prime }\left(u\right)=2\int_{0}^{u}K_{h}\left(v\right)\;dv$ with $K_{h}\left(v\right)=K\left(v/h\right)/h$. By Assumptions 1 and 3, we have

$$\begin{array}{c} \max_{i,j\in [N],k\in [p]}\left|L_{h}^{\prime }\left(\varepsilon_{i}-\varepsilon_{j}\right)\left(X_{i,k}-X_{j,k}\right)\right|\leq C_{1}\;. \end{array}$$

Moreover, by Lemma 1 of Zhou et al. [17] and the fact that the distribution of $\varepsilon_{i}-\varepsilon_{j}$ is symmetric about zero, we have $E\{\nabla {\hat{L}}_{h}\left(\beta^{*}\right)\}=0$. Then, by Bonferroni inequality and Equation (A.1) of Clémençon et al. [25], it holds that

$$\begin{array}{cc} P(|\nabla {\hat{L}}_{h}\left(\beta^{*}\right){|}_{\infty }>x)\leq & \;p\max_{k\in [p]}P\left\{|\frac{1}{N(N-1)}\sum_{i=1}^{N}\sum_{j\neq i}L_{h}^{\prime }\left(\varepsilon_{i}-\varepsilon_{j}\right)\left(X_{i,k}-X_{j,k}\right)|>x\right\}\\ \leq & \;2p\;\exp(-C_{1}nx^{2}) \end{array}$$

for any x>0, which implies that

$$\begin{array}{c} |\nabla {\hat{L}}_{h}\left(\beta^{*}\right){|}_{\infty }≲\frac{{\left(\;\log\;p+u\right)}^{1/2}}{N^{1/2}} \end{array}$$

for any u>0, with probability at least $1-e^{-u}$. Then, we complete the proof of Lemma A2. □

### Appendix C.3. Proof of Lemma A3

**Proof of** **Lemma A3.** For any $\beta \in R^{d}$, write $\overset{ˇ}{\beta }=\left(\beta -\beta^{*}\right)/{\left|\beta -\beta^{*}\right|}_{\Sigma }$. Consider the event

$$\begin{array}{c} A_{i,j}\left(\beta \right)=\left\{\right|\varepsilon_{i}-\varepsilon_{j}\left|\leq b/2\}\cap \{\right|{\left(X_{i}-X_{j}\right)}^{⊤}\overset{ˇ}{\beta }\left|\leq b/\left(2r\right)\right\}\;. \end{array}$$

Restricted on $A_{i,j}\left(\beta \right)$, for any $\beta \in B_{\Sigma }\left(\beta^{*},r\right)$, we have

$$\begin{array}{cc} |\left(Y_{i}-X_{i}^{⊤}\beta \right)-\left(Y_{j}-X_{j}^{⊤}\beta \right)|= & \;|\left(Y_{i}-X_{i}^{⊤}\beta^{*}\right)+X_{i}^{⊤}\left(\beta^{*}-\beta \right)-\left(Y_{j}-X_{j}^{⊤}\beta^{*}\right)+X_{j}^{⊤}\left(\beta -\beta^{*}\right)|\\ \leq & \;|\varepsilon_{i}-\varepsilon_{j}\left|+\right|X_{i}^{⊤}\left(\beta -\beta^{*}\right)\left|+\right|X_{j}^{⊤}\left(\beta -\beta^{*}\right)|\\ \leq & \;b/2+b/2=b\;. \end{array}$$

By Assumption 3,

(A23) $$\begin{array}{cc} \; & \langle \nabla {\hat{L}}_{h}\left(\beta \right)-\nabla {\hat{L}}_{h}\left(\beta^{*}\right),\beta -\beta^{*}\rangle \\ \; & \;\;\;=\langle \nabla {\hat{L}}_{1,h}\left(\beta \right)-\nabla {\hat{L}}_{1,h}\left(\beta^{*}\right),\beta -\beta^{*}\rangle \\ \; & \;\;\;=\frac{1}{n(n-1)}\sum_{i\in I_{1}}\sum_{j\neq i\in I_{1}}\left\{L_{1,h}^{\prime }\left(\varepsilon_{i}\left(\beta \right)-\varepsilon_{j}\left(\beta \right)\right)-L_{1,h}^{\prime }\left(\varepsilon_{i}-\varepsilon_{j}\right)\right\}\langle X_{j}-X_{i},\beta -\beta^{*}\rangle \\ \; & \;\;\;\geq \frac{1}{n(n-1)}\sum_{i\in I_{1}}\sum_{j\neq i\in I_{1}}\left\{L_{1,h}^{\prime }\left(\varepsilon_{i}\left(\beta \right)-\varepsilon_{j}\left(\beta \right)\right)-L_{1,h}^{\prime }\left(\varepsilon_{i}-\varepsilon_{j}\right)\right\}\langle X_{j}-X_{i},\beta -\beta^{*}\rangle I\left\{A_{i,j}\left(\beta \right)\right\}\\ \; & \;\;\;\geq \frac{2k_{5}}{n(n-1)b}\sum_{i\in I_{1}}\sum_{j\neq i\in I_{1}}{\left(\langle X_{j}-X_{i},\beta -\beta^{*}\rangle \right)}^{2}I\left\{A_{i,j}\left(\beta \right)\right\} \end{array}$$

with $\varepsilon_{i}\left(\beta \right)=Y_{i}-X_{i}^{⊤}\beta$. For any R>0, define the functions

(A24) $$\begin{array}{cc} \varphi_{R}\left(x\right) & =x^{2}I\left(|x|\leq R/2\right)+{\left\{x-\mathrm{sign}\left(x\right)\cdot R\right\}}^{2}I\left(R/2<|x|\leq R\right)\;, \end{array}$$

which are smoothed proxies of $x\to x^{2}I\left(|x|\leq R\right)$. Moreover, $\varphi_{R}\left(\cdot \right)$ is *R*-Lipschitz continuous and satisfies (i) $x^{2}I\left(|x|\leq R/2\right)\leq \varphi_{R}\left(x\right)\leq x^{2}I(|x|\leq R)\leq R|x|$ and (ii) $\varphi_{cR}\left(cx\right)=c^{2}\varphi_{R}\left(x\right)$ for any c>0. Combining (A23), we have

(A25) $$\begin{array}{cc} \; & \langle \nabla {\hat{L}}_{h}\left(\beta \right)-\nabla {\hat{L}}_{h}\left(\beta^{*}\right),\beta -\beta^{*}\rangle \\ \; & \;\;\;\geq \frac{2k_{5}}{n(n-1)b}\sum_{i\in I_{1}}\sum_{j\neq i\in I_{1}}\varphi_{b|\beta -\beta^{*}{|}_{\Sigma }/\left(2r\right)}\left({\left(X_{i}-X_{j}\right)}^{⊤}\left(\beta -\beta^{*}\right)\right)I(|\varepsilon_{i}-\varepsilon_{j}\left|\leq b/2\right)\\ \; & \;\;\;\geq 2k_{5}{\left|\beta -\beta^{*}\right|}_{\Sigma }\times \underset{U(\overset{ˇ}{\beta })}{\underset{︸}{\frac{1}{n(n-1)b}\sum_{i\in I_{1}}\sum_{j\neq i\in I_{1}}\varphi_{b/(2r)}\left({\left(X_{i}-X_{j}\right)}^{⊤}\overset{ˇ}{\beta }\right)I(|\varepsilon_{i}-\varepsilon_{j}\left|\leq b/2\right)}} \end{array}$$

with $\overset{ˇ}{\beta }=\left(\beta -\beta^{*}\right)/{\left|\beta -\beta^{*}\right|}_{\Sigma }$. Recall $\Theta \left(r,l\right)=\{\beta \in R^{p}:\beta -\beta^{*}\in B_{\Sigma }\left(r\right)\cap C_{\Sigma }\left(l\right)\}$. As we will show in Appendix C.4 and Appendix C.5,

(A26) $$\begin{array}{c} \underset{\beta \in \Theta (r,l)}{\inf}E\left\{U\left(\overset{ˇ}{\beta }\right)\right\}\geq \frac{7}{8k_{5}} \end{array}$$

provided that $b\leq {\left(2k_{4}k_{5}\right)}^{-1}$ and $r\leq {\left(256k_{2}\right)}^{-1/2}h$, and

(A27) $$\begin{array}{c} \underset{\beta -\beta^{*}\in C_{\Sigma }\left(l\right)}{\sup}\left|\left(1-E\right)\left\{U\left(\overset{ˇ}{\beta }\right)\right\}\right|\leq \frac{8k_{1}l{\left(\;\log\;p\right)}^{1/2}}{rn^{1/2}}+\frac{7h}{16k_{5}r}\times {\left(\frac{2u}{n}\right)}^{1/2}+\frac{5hu}{6n} \end{array}$$

with probability at least $1-e^{-u}$ for any u>0. Taking u=logp, by (A25), we have

$$\begin{array}{cc} \frac{\langle \nabla {\hat{L}}_{h}\left(\beta \right)-\nabla {\hat{L}}_{h}\left(\beta^{*}\right),\beta -\beta^{*}\rangle }{|\beta -\beta^{*}{|}_{\Sigma }}\geq & \;2k_{5}U\left(\overset{ˇ}{\beta }\right)\\ \geq & \;2k_{5}\underset{\beta \in \Theta (r,l)}{\inf}E\left\{U\left(\overset{ˇ}{\beta }\right)\right\}-\underset{\beta -\beta^{*}\in C_{\Sigma }\left(l\right)}{\sup}\left|\left(1-E\right)\left\{U\left(\overset{ˇ}{\beta }\right)\right\}\right|\\ \geq & \;\frac{7}{4}-\frac{16k_{1}k_{5}l{\left(\;\log\;p\right)}^{1/2}}{rn^{1/2}}-\frac{7h}{8r}\times {\left(\frac{2\;\log\;p}{n}\right)}^{1/2}-\frac{10k_{5}h\;\log\;p}{6n}\\ \geq & \;\frac{7}{8} \end{array}$$

provided that $h\leq {\left(2k_{4}k_{5}\right)}^{-1}$ and $r\leq {\left(256k_{2}\right)}^{-1/2}h$ and

$$\begin{array}{c} n\geq \max\left\{\frac{{\left(55k_{1}k_{5}l\right)}^{2}\;\log\;p}{r^{2}},\frac{18h^{2}\;\log\;p}{r^{2}},\frac{40k_{5}h\;\log\;p}{7}\right\}\;, \end{array}$$

with probability at least $1-p^{-1}$. Then, we complete the proof of Lemma A3. □

### Appendix C.4. Proof of (A26)

**Proof** **of** **(A26).** By Assumption 2, we have

$$\begin{array}{cc} E\{I(|\varepsilon_{i}-\varepsilon_{j}|\leq h/2)\;|\;X_{i},X_{j}\}= & \;\int_{-h/2}^{h/2}g\left(u\right)\;du=hg\left(0\right)+\int_{-h/2}^{h/2}\left\{g\left(u\right)-g\left(0\right)\right\}\;du\\ \geq & \;hg\left(0\right)-\int_{-h/2}^{h/2}|g\left(u\right)-g\left(0\right)|\;du\geq h/k_{5}-k_{4}\int_{-h/2}^{h/2}\left|u\right|\;du\\ \geq & \;h/k_{5}-k_{4}h^{2}/4\geq \frac{7h}{8k_{5}} \end{array}$$

provided that $h\leq {\left(2k_{4}k_{5}\right)}^{-1}$. Hence, by $x^{2}I\left(|x|\leq R/2\right)\leq \varphi_{R}\left(x\right)$, we have

(A28) $$\begin{array}{cc} E\{U\left(\overset{ˇ}{\beta }\right)\}\geq & \;\frac{1}{n(n-1)h}\sum_{i\in I_{1}}\sum_{j\neq i\in I_{1}}E[{\left\{{\left(X_{i}-X_{j}\right)}^{⊤}\overset{ˇ}{\beta }\right\}}^{2}\\ \; & \;\;\;\;\;\;\;\;\;\;\;\;\;\;\;\;\;\;\;\;\;\;\;\;\;\;\;\;\;\;\;\;\times I\{|{\left(X_{i}-X_{j}\right)}^{⊤}\overset{ˇ}{\beta }|\leq h/\left(4r\right)\}I(|\varepsilon_{i}-\varepsilon_{j}\left|\leq h/2\right)]\\ \geq & \;\frac{7}{8k_{5}n\left(n-1\right)}\sum_{i\in I_{1}}\sum_{j\neq i\in I_{1}}E[{\left\{{\left(X_{i}-X_{j}\right)}^{⊤}\overset{ˇ}{\beta }\right\}}^{2}I\{|{\left(X_{i}-X_{j}\right)}^{⊤}\overset{ˇ}{\beta }|\leq h/\left(4r\right)\}] \end{array}$$

provided that $h\leq {\left(2k_{4}k_{5}\right)}^{-1}$. Notice that

(A29) $$\begin{array}{cc} \; & E[{\left\{{\left(X_{i}-X_{j}\right)}^{⊤}\overset{ˇ}{\beta }\right\}}^{2}I(|{\left(X_{i}-X_{j}\right)}^{⊤}\overset{ˇ}{\beta }|\leq h/\left(4r\right))]\\ \; & \;\;\;=E\left[{\left\{{\left(X_{i}-X_{j}\right)}^{⊤}\overset{ˇ}{\beta }\right\}}^{2}\right]-E[{\left\{{\left(X_{i}-X_{j}\right)}^{⊤}\overset{ˇ}{\beta }\right\}}^{2}I(|{\left(X_{i}-X_{j}\right)}^{⊤}\overset{ˇ}{\beta }|>h/\left(4r\right))]\;. \end{array}$$

We have

(A30) $$\begin{array}{cc} E[{\left\{{\left(X_{i}-X_{j}\right)}^{⊤}\overset{ˇ}{\beta }\right\}}^{2}]= & \;E\left[{\left\{{\left(X_{i}-X_{j}\right)}^{⊤}\left(\beta -\beta^{*}\right)\right\}}^{2}\right]/{\left|\beta -\beta^{*}\right|}_{\Sigma }^{2}\\ = & \;{\left(\beta -\beta^{*}\right)}^{⊤}E\left(X_{i}X_{i}^{⊤}-X_{i}X_{j}^{⊤}-X_{j}X_{i}^{⊤}+X_{j}X_{j}^{⊤}\right)\left(\beta -\beta^{*}\right)/{\left|\beta -\beta^{*}\right|}_{\Sigma }^{2}\\ = & \;{\left(\beta -\beta^{*}\right)}^{⊤}\left\{E\left(X_{i}X_{i}^{⊤}\right)+E\left(X_{j}X_{j}^{⊤}\right)\right\}{\left(\beta -\beta^{*}\right)}^{⊤}/{\left|\beta -\beta^{*}\right|}_{\Sigma }^{2}=2\;. \end{array}$$

By Assumption 1 and Markov inequality, it holds that

$$\begin{array}{cc} \; & E[{\left\{{\left(X_{i}-X_{j}\right)}^{⊤}\overset{ˇ}{\beta }\right\}}^{2}I(|{\left(X_{i}-X_{j}\right)}^{⊤}\overset{ˇ}{\beta }|>h/\left(4r\right))]\\ \; & \;\;\;\leq {\left(E\left[{\left\{{\left(X_{i}-X_{j}\right)}^{⊤}\overset{ˇ}{\beta }\right\}}^{4}\right]\right)}^{1/2}\left[P\{\right|{\left(X_{i}-X_{j}\right)}^{⊤}\overset{ˇ}{\beta }|>h/\left(4r\right){\}]}^{1/2}\\ \; & \;\;\;\leq 16{\left(r/h\right)}^{2}E\left[{\left\{{\left(X_{i}-X_{j}\right)}^{⊤}\overset{ˇ}{\beta }\right\}}^{4}\right]\\ \; & \;\;\;\leq 16{\left(r/h\right)}^{2}\underset{u\in S^{p-1}}{\sup}E\left[{\left\{{\left(X_{i}-X_{j}\right)}^{⊤}u\right\}}^{4}\right]\\ \; & \;\;\;\leq 256{\left(r/h\right)}^{2}\underset{u\in S^{p-1}}{\sup}E\left\{{\left(X^{⊤}u\right)}^{4}\right\}\\ \; & \;\;\;\leq 256k_{2}{\left(r/h\right)}^{2}\;. \end{array}$$

Together with (A29) and (A30), by (A28), we have

$$\begin{array}{c} E\left\{U\left(\overset{ˇ}{\beta }\right)\right\}\geq \frac{7\{2-256k_{2}{\left(r/h\right)}^{2}\}}{8k_{5}}\geq \frac{7}{8k_{5}} \end{array}$$

provided that $b\leq {\left(2k_{4}k_{5}\right)}^{-1}$ and $r\leq {\left(256k_{2}\right)}^{-1/2}h$. Then, (A26) holds. □

### Appendix C.5. Proof of (A27)

**Proof** **of** **(A27).** Write $\varpi \left(X_{i},X_{j},\delta \right)=\varphi_{h/(2r)}({\left(X_{i}-X_{j}\right)}^{⊤}\delta /{\left|\delta \right|}_{\Sigma })I(|\varepsilon_{i}-\varepsilon_{j}\left|\leq h/2\right)/h$. Since $|\varphi_{R}\left(x\right)|\leq R|x|I(|x|\leq R)\leq R^{2}$, we have $|\varpi (X_{i},X_{j},\delta )|\leq h/4$ and

$$\begin{array}{cc} E\{\varpi^{2}\left(X_{i},X_{j},\delta \right)\}\leq & \;\frac{1}{4}E[\{{\left(X_{i}-X_{j}\right)}^{⊤}\delta /{\left|\delta \right|}_{\Sigma }{\}}^{2}E\{I(|\varepsilon_{i}-\varepsilon_{j}|\leq h/2)\;|\;X_{i},X_{j}\}]\\ \leq & \;\frac{7h}{32k_{5}}\delta E\left\{\left(X_{i}-X_{j}\right){\left(X_{i}-X_{j}\right)}^{⊤}\right\}\delta /{\left|\delta \right|}_{\Sigma }^{2}\\ \leq & \;\frac{7h}{16k_{5}r}\;. \end{array}$$

By Theorem 2 of Rejchel [24], we have

(A31) $$\begin{array}{cc} \underset{\beta -\beta^{*}\in C_{\Sigma }\left(l\right)}{\sup}\left|\left(1-E\right)\left\{U\left(\overset{ˇ}{\beta }\right)\right\}\right|= & \;\underset{\delta \in C_{\Sigma }\left(l\right)}{\sup}|\frac{1}{n(n-1)}\sum_{i\in I_{1}}\sum_{j\neq i\in I_{1}}\left(1-E\right)\left\{\varpi \left(X_{i},X_{j},\delta \right)\right\}|\\ \leq & \;4\underset{U_{1}}{\underset{︸}{E\left\{\sup_{\delta \in C_{\Sigma }\left(l\right)}|\frac{1}{\left\lfloor n/2\right\rfloor }\sum_{i=1}^{\left\lfloor n/2\right\rfloor }\sigma_{i}\varpi \left(X_{i},X_{\lfloor n/2\rfloor +i},\delta \right)|\right\}}}\\ \; & +\frac{7b}{16k_{5}r}\times {\left(\frac{2u}{n}\right)}^{1/2}+\frac{5bu}{6n} \end{array}$$

with probability at least $1-e^{-u}$ for any u>0, where ${\left\{\sigma_{i}\right\}}_{i=1}^{\left\lfloor n/2\right\rfloor }$ is a sequence of Rademacher random variables. Write $X_{i}={\left(X_{i,1},\ldots ,X_{i,p}\right)}^{⊤}$. Due to $\psi_{b/(2r)}\left(x\right)/b$ being a 1/(2r)-Lipschitz function, by Talagrand’s contraction principle, we have

$$\begin{array}{cc} U_{1}\leq & \;\frac{1}{r}E\left\{\underset{\delta \in C_{\Sigma }\left(l\right)}{\sup}|\frac{1}{\left\lfloor n/2\right\rfloor }\sum_{i=1}^{\left\lfloor n/2\right\rfloor }\sigma_{i}{\left(X_{i}-X_{\lfloor n/2\rfloor +i}\right)}^{⊤}\delta I(|\varepsilon_{i}-\varepsilon_{\lfloor n/2\rfloor +i}{\left|\leq b/2)/|\delta \right|}_{\Sigma }|\right\}\\ \leq & \;\frac{l}{r}E\left\{|\frac{1}{\left\lfloor n/2\right\rfloor }\sum_{i=1}^{\left\lfloor n/2\right\rfloor }\sigma_{i}\left(X_{i}-X_{\lfloor n/2\rfloor +i}\right)I(|\varepsilon_{i}-\varepsilon_{\lfloor n/2\rfloor +i}\left|\leq b/2\right){|}_{\infty }\right\}\\ = & \;\frac{l}{r}E\left\{\max_{k\in [p]}|\frac{1}{\left\lfloor n/2\right\rfloor }\sum_{i=1}^{\left\lfloor n/2\right\rfloor }\sigma_{i}\left(X_{i,k}-X_{\lfloor n/2\rfloor +i,k}\right)I(|\varepsilon_{i}-\varepsilon_{\lfloor n/2\rfloor +i}\left|\leq b/2\right)|\right\}\\ \leq & \;\frac{2k_{1}l{\left(\;\log\;p\right)}^{1/2}}{r{\left(\left\lfloor n/2\right\rfloor \right)}^{1/2}}\leq \frac{8k_{1}l{\left(\;\log\;p\right)}^{1/2}}{rn^{1/2}}\;, \end{array}$$

where the last inequality is due to $\sigma_{i}\left(X_{i,k}-X_{\lfloor n/2\rfloor +i,k}\right)I\{|\varepsilon_{\pi (i)}-\varepsilon_{\pi (\lfloor n/2\rfloor +i)}\left|\leq b/2\right\}$ being a sub-Gaussian random variable for any i∈[⌊n/2⌋] and k∈[p]. By (A31), it holds that

$$\begin{array}{c} \underset{\beta -\beta^{*}\in C_{\Sigma }\left(l\right)}{\sup}\left|\left(1-E\right)\left\{U\left(\overset{ˇ}{\beta }\right)\right\}\right|\leq \frac{8k_{1}l{\left(\;\log\;p\right)}^{1/2}}{rn^{1/2}}+\frac{7b}{16k_{5}r}\times {\left(\frac{2u}{n}\right)}^{1/2}+\frac{5bu}{6n} \end{array}$$

with probability at least $1-e^{-u}$ for any u>0. Hence, (A27) holds. □

### Appendix C.6. Proof of Lemma A4

**Proof of** **Lemma A4.** Let $\tilde{\mu }={\hat{\beta }}^{\left(0\right)}-\beta^{*}$. As we show in Appendix C.7 that

(A32) $$\begin{array}{c} |\tilde{\mu }{|}_{\Sigma }\leq r_{0} \end{array}$$

with probability at least $1-p^{-1}$, provided that $16k_{2}^{1/2}r_{1}\leq h\leq {\left(2k_{4}k_{5}\right)}^{-1}$, $\tau_{1}r_{1}^{2}\geq 7{\left(55k_{1}k_{5}\right)}^{2}/8$ and ${\left(18h^{2}\;\log\;p/n\right)}^{1/2}\leq r_{1}\leq {\left(32k_{2}^{1/2}k_{4}k_{5}\right)}^{-1}$ and

(A33) $$\begin{array}{c} R\leq \min\left\{\frac{7r_{1}^{2}}{8k_{8}\lambda_{0}},\frac{{\tilde{C}}_{1}r_{1}^{2}n^{1/2}}{{\left(\;\log\;p\right)}^{1/2}},{\left(\frac{7r_{1}^{2}n}{8\tau_{1}\;\log\;p}\right)}^{1/2}\right\}\;. \end{array}$$

Then, by (A2) and (A32), we have

$$\begin{array}{c} \langle \nabla {\hat{L}}_{1,h}\left({\hat{\beta }}^{\left(0\right)}\right)-\nabla {\hat{L}}_{1,h}\left(\beta^{*}\right),\tilde{\mu }\rangle =\langle \nabla {\hat{L}}_{h}\left({\hat{\beta }}^{\left(0\right)}\right)-\nabla {\hat{L}}_{h}\left(\beta^{*}\right),\tilde{\mu }\rangle \geq \frac{7|\tilde{\mu }{|}_{\Sigma }^{2}}{8}-\frac{\tau_{1}\;\log\;p{\left|\tilde{\mu }\right|}_{1}^{2}}{n} \end{array}$$

with probability at least $1-2p^{-1}$, provided that $16k_{2}^{1/2}r_{1}\leq h\leq {\left(2k_{4}k_{5}\right)}^{-1}$, $\tau_{1}r_{1}^{2}\geq 7{\left(55k_{1}k_{5}\right)}^{2}/8$ and ${\left(18h^{2}\;\log\;p/n\right)}^{1/2}\leq r_{1}\leq {\left(32k_{2}^{1/2}k_{4}k_{5}\right)}^{-1}$ and *R* satisfies (A33). Write

$$\begin{array}{c} {\tilde{\Pi }}_{3}={\left|\nabla {\hat{L}}_{1,h}\left(\beta^{*}\right)\right|}_{\infty }\;. \end{array}$$

Analogously to the proof of (A10), we have

$$\begin{array}{cc} \frac{7-4k_{3}\mu }{8}\cdot {\left|\tilde{\mu }\right|}_{\Sigma }^{2}\leq & \;p_{\lambda_{0}}\left(\beta^{*}\right)-p_{\lambda_{0}}\left({\hat{\beta }}^{\left(0\right)}\right)-\langle \nabla {\hat{L}}_{1,h}\left(\beta^{*}\right),\tilde{\mu }\rangle +\frac{\tau_{1}\;\log\;p{\left|\tilde{\mu }\right|}_{1}^{2}}{n}\\ \leq & \;p_{\lambda_{0}}\left(\beta^{*}\right)-p_{\lambda_{0}}\left({\hat{\beta }}^{\left(0\right)}\right)+{\tilde{\Pi }}_{3}\cdot {\left|\tilde{\mu }\right|}_{1}+\frac{\tau_{1}\;\log\;p{\left|\tilde{\mu }\right|}_{1}^{2}}{n}\\ \leq & \;p_{\lambda_{0}}\left(\beta^{*}\right)-p_{\lambda_{0}}\left({\hat{\beta }}^{\left(0\right)}\right)+\left\{{\tilde{\Pi }}_{3}+\frac{2R\tau_{1}\;\log\;p}{n}\right\}\cdot {\left|\tilde{\mu }\right|}_{1} \end{array}$$

with probability at least $1-2p^{-1}$, provided that $16k_{2}^{1/2}r_{1}\leq h\leq {\left(2k_{4}k_{5}\right)}^{-1}$, $\tau_{1}r_{1}^{2}\geq 7{\left(55k_{1}k_{5}\right)}^{2}/8$ and ${\left(18h^{2}\;\log\;p/n\right)}^{1/2}\leq r_{1}\leq {\left(32k_{2}^{1/2}k_{4}k_{5}\right)}^{-1}$ and *R* satisfies (A33). Let $\lambda_{0}$ satisfy that

$$\begin{array}{c} \lambda_{0}\geq 4k_{8}^{-1}\left\{{\tilde{\Pi }}_{3}+\frac{2R\tau_{1}\;\log\;p}{n}\right\}\;. \end{array}$$

Recall $0<\mu <7/(5k_{3})$. Analogously to the deviation (A11), it holds that

$$\begin{array}{c} 0\leq \frac{7-5k_{3}\mu }{8}\cdot {\left|\tilde{\mu }\right|}_{\Sigma }^{2}\leq \frac{3p_{\lambda_{0}}\left(\beta^{*}\right)}{2}-\frac{p_{\lambda_{0}}\left({\hat{\beta }}^{\left(0\right)}\right)}{2} \end{array}$$

with probability at least $1-2p^{-1}$, provided that $16k_{2}^{1/2}r_{1}\leq h\leq {\left(2k_{4}k_{5}\right)}^{-1}$, $\tau_{1}r_{1}^{2}\geq 7{\left(55k_{1}k_{5}\right)}^{2}/8$ and ${\left(18h^{2}\;\log\;p/n\right)}^{1/2}\leq r_{1}\leq {\left(32k_{2}^{1/2}k_{4}k_{5}\right)}^{-1}$ and *R* satisfies (A33). Recall $S=\mathrm{supp}\left(\beta^{*}\right)\subseteq \left[p\right]$ can be the true active set with cardinality |S|≤s. By Lemma A.5 of Loh and Wainwright [21], we have

$$\begin{array}{c} 0\leq 3p_{\lambda_{0}}\left(\beta^{*}\right)-p_{\lambda_{0}}\left({\hat{\beta }}^{\left(0\right)}\right)\leq 3k_{8}\lambda_{0}|{\tilde{\mu }}_{S}{|}_{1}-k_{8}\lambda_{0}{\left|{\tilde{\mu }}_{S^{c}}\right|}_{1}\;, \end{array}$$

which implies that $|{\tilde{\mu }}_{S^{c}}{|}_{1}\leq 3{\left|{\tilde{\mu }}_{S}\right|}_{1}$, with probability at least $1-2p^{-1}$, provided that $16k_{2}^{1/2}r_{1}\leq h\leq {\left(2k_{4}k_{5}\right)}^{-1}$, $\tau_{1}r_{1}^{2}\geq 7{\left(55k_{1}k_{5}\right)}^{2}/8$ and ${\left(18h^{2}\;\log\;p/n\right)}^{1/2}\leq r_{1}\leq {\left(32k_{2}^{1/2}k_{4}k_{5}\right)}^{-1}$ and *R* satisfies (A33). Hence,

$$\begin{array}{c} |\tilde{\mu }{|}_{1}=|{\tilde{\mu }}_{S}{|}_{1}+|{\tilde{\mu }}_{S^{c}}{|}_{1}\leq 4s^{1/2}{\left|{\tilde{\mu }}_{S}\right|}_{2}\;, \end{array}$$

with probability at least $1-2p^{-1}$, provided that $16k_{2}^{1/2}r_{1}\leq h\leq {\left(2k_{4}k_{5}\right)}^{-1}$, $\tau_{1}r_{1}^{2}\geq 7{\left(55k_{1}k_{5}\right)}^{2}/8$ and ${\left(18h^{2}\;\log\;p/n\right)}^{1/2}\leq r_{1}\leq {\left(32k_{2}^{1/2}k_{4}k_{5}\right)}^{-1}$ and *R* satisfies (A33). Then, we obtain that $\left\{{\hat{\beta }}^{\left(0\right)}\in \Lambda \right\}$ with probability at least $1-2p^{-1}$, provided that $16k_{2}^{1/2}r_{1}\leq h\leq {\left(2k_{4}k_{5}\right)}^{-1}$, $\tau_{1}r_{1}^{2}\geq 7{\left(55k_{1}k_{5}\right)}^{2}/8$ and ${\left(18h^{2}\;\log\;p/n\right)}^{1/2}\leq r_{1}\leq {\left(32k_{2}^{1/2}k_{4}k_{5}\right)}^{-1}$ and *R* satisfies (A33). Analogous to the proof of (A13), we have

(A34) $$\begin{array}{c} |\tilde{\mu }{|}_{\Sigma }≲s^{1/2}\lambda_{0} \end{array}$$

with probability at least $1-2p^{-1}$, provided that $16k_{2}^{1/2}r_{1}\leq h\leq {\left(2k_{4}k_{5}\right)}^{-1}$, $\tau_{1}r_{1}^{2}\geq 7{\left(55k_{1}k_{5}\right)}^{2}/8$ and ${\left(18h^{2}\;\log\;p/n\right)}^{1/2}\leq r_{1}\leq {\left(32k_{2}^{1/2}k_{4}k_{5}\right)}^{-1}$ and *R* satisfies (A33). Analogous to the proof of Lemma A2, we can show that

(A35) $$\begin{array}{c} {\tilde{\Pi }}_{3}≲\frac{{\left(\;\log\;p+u\right)}^{1/2}}{n^{1/2}} \end{array}$$

for any u>0, with probability at least $1-e^{-u}$. To ensure that $|{\hat{\beta }}^{\left(0\right)}-\beta^{*}{|}_{\Sigma }$ converges as fast as possible, selecting $r_{1}=O\left(1\right)$, $\tau_{1}=O\left(1\right)$, h=O(1), $R≍n^{1/2}{\left(\;\log\;p\right)}^{-1/2}$ and

$$\begin{array}{c} \lambda_{0}≍\frac{{\left(\;\log\;p\right)}^{1/2}}{n^{1/2}}\;. \end{array}$$

Together with (A34), it holds that

$$\begin{array}{c} |{\hat{\beta }}^{\left(0\right)}-\beta^{*}{|}_{\Sigma }≲\frac{{\left(s\;\log\;p\right)}^{1/2}}{n^{1/2}}\;, \end{array}$$

provided that h=O(1), $R≍{\left(n/\;\log\;p\right)}^{1/2}$ and $\lambda_{0}≍{\left(\;\log\;p/n\right)}^{1/2}$. Then, we complete the proof of Lemma A4. □

### Appendix C.7. Proof of (A32)

**Proof** **of** **(A32).** Notice that If $|\tilde{\mu }{|}_{\Sigma }>r_{0}$, by $\nabla {\hat{L}}_{1,h}\left(\beta^{*}+\tilde{\mu }\right)=-\nabla p_{\lambda_{0}}\left({\hat{\beta }}^{\left(0\right)}\right)$ and (A3), we have

(A36) $$\begin{array}{cc} \langle -\nabla p_{\lambda_{0}}\left({\hat{\beta }}^{\left(0\right)}\right)-\nabla {\hat{L}}_{1,h}\left(\beta^{*}\right),\mu \rangle = & \;\langle \nabla {\hat{L}}_{1,h}\left(\beta^{*}+\tilde{\mu }\right)-\nabla {\hat{L}}_{1,h}\left(\beta^{*}\right),\tilde{\mu }\rangle \\ = & \;\langle \nabla {\hat{L}}_{h}\left(\beta^{*}+\tilde{\mu }\right)-\nabla {\hat{L}}_{h}\left(\beta^{*}\right),\tilde{\mu }\rangle \\ \geq & \;\frac{7r_{1}{\left|\tilde{\mu }\right|}_{\Sigma }}{8}-\frac{2R\tau_{1}\;\log\;p{\left|\tilde{\mu }\right|}_{1}}{n} \end{array}$$

provided that $16k_{2}^{1/2}r_{1}\leq h\leq {\left(2k_{4}k_{5}\right)}^{-1}$, $\tau_{1}r_{1}^{2}\geq 7{\left(55k_{1}k_{5}\right)}^{2}/8$ and ${\left(18h^{2}\;\log\;p/n\right)}^{1/2}\leq r_{1}\leq {\left(32k_{2}^{1/2}k_{4}k_{5}\right)}^{-1}$, with probability at least $1-p^{-1}$. By Assumption 4, the Hölder inequality and Lemma 4 of Loh and Wainwright [21], we have

$$\begin{array}{cc} \langle -\nabla p_{\lambda_{0}}\left({\hat{\beta }}^{\left(0\right)}\right)-\nabla {\hat{L}}_{1,h}\left(\beta^{*}\right),\tilde{\mu }\rangle \leq & \;\{|\nabla p_{\lambda_{0}}\left({\hat{\beta }}^{\left(0\right)}\right){|}_{\infty }+|\nabla {\hat{L}}_{1,h}\left(\beta^{*}\right){|}_{\infty }\}\cdot |\tilde{\mu }{|}_{1}\\ \leq & \;\left(k_{8}\lambda_{0}+{\tilde{\Pi }}_{3}\right){\left|\tilde{\mu }\right|}_{1}\;. \end{array}$$

Recall ${\tilde{\Pi }}_{3}={\left|\nabla {\hat{L}}_{1,h}\left(\beta^{*}\right)\right|}_{\infty }$. and $|\tilde{\mu }{|}_{1}\leq 2R$. Together with (A35) and (A36), selecting u=2logp, we have

$$\begin{array}{c} \frac{7r_{1}{\left|\tilde{\mu }\right|}_{\Sigma }}{8}\leq \left\{k_{8}\lambda_{0}+\frac{C_{1}{\left(\;\log\;p\right)}^{1/2}}{n^{1/2}}+\frac{2R\tau_{1}\;\log\;p}{n}\right\}R \end{array}$$

which implies that

$$\begin{array}{c} |\tilde{\mu }{|}_{\Sigma }\leq \frac{8}{7r_{1}}\left\{k_{8}\lambda_{0}+\frac{C_{1}{\left(\;\log\;p\right)}^{1/2}}{n^{1/2}}+\frac{2R\tau_{1}\;\log\;p}{n}\right\}R\leq r_{1} \end{array}$$

with probability at least $1-2p^{-1}$, provided that $16k_{2}^{1/2}r_{1}\leq h\leq {\left(2k_{4}k_{5}\right)}^{-1}$, $\tau_{1}r_{1}^{2}\geq 7{\left(55k_{1}k_{5}\right)}^{2}/8$ and ${\left(18h^{2}\;\log\;p/n\right)}^{1/2}\leq r_{1}\leq {\left(32k_{2}^{1/2}k_{4}k_{5}\right)}^{-1}$ and

$$\begin{array}{c} R\leq \min\left\{\frac{7r_{1}^{2}}{8k_{8}\lambda_{0}},\frac{{\tilde{C}}_{1}r_{1}^{2}n^{1/2}}{{\left(\;\log\;p\right)}^{1/2}},{\left(\frac{7r_{1}^{2}n}{8\tau_{1}\;\log\;p}\right)}^{1/2}\right\}\;, \end{array}$$

where ${\tilde{C}}_{1}>0$ is a sufficiently small constant. Hence, (A32) holds. □
